# Equilibrium States and Their Stability in the Head-Direction Ring Network

**DOI:** 10.3389/fncom.2019.00096

**Published:** 2020-01-23

**Authors:** Caixia Wang, Kechen Zhang

**Affiliations:** ^1^School of International Economics, China Foreign Affairs University, Beijing, China; ^2^Department of Biomedical Engineering, Johns Hopkins University School of Medicine, Baltimore, MD, United States

**Keywords:** continuous attractor network, ring network, neural field, dynamical system, Fourier analysis, stability

## Abstract

Head-direction cells have been found in several areas in the mammalian brains. The firing rate of an ideal head-direction cell reaches its peak value only when the animal's head points in a specific direction, and this preferred direction stays the same regardless of spatial location. In this paper we combine mathematical analytical techniques and numerical simulations to fully analyze the equilibrium states of a generic ring attractor network, which is a widely used modeling framework for the head-direction system. Under specific conditions, all solutions of the ring network are bounded, and there exists a Lyapunov function that guarantees the stability of the network for any given inputs, which may come from multiple sources in the biological system, including self-motion information for inertially based updating and landmark information for calibration. We focus on the first few terms of the Fourier series of the ring network to explicitly solve for all possible equilibrium states, followed by a stability analysis based on small perturbations. In particular, these equilibrium states include the standard single-peaked activity pattern as well as double-peaked activity pattern, whose existence is unknown but has testable experimental implications. To our surprise, we have also found an asymmetric equilibrium activity profile even when the network connectivity is strictly symmetric. Finally we examine how these different equilibrium solutions depend on the network parameters and obtain the phase diagrams in the parameter space of the ring network.

## 1. Introduction

Head-direction cells were first reported in several brain areas related to the limbic system in the rodents (Taube, 2007) and later in other mammalian species such as monkeys (Robertson et al., 1999) and bats (Finkelstein et al., 2015). A stereotypical head-direction cell increases its firing rate when the animal's head is facing in a specific direction in a world-centered coordinate system regardless of the animal's spatial location, and the firing rate decreases to its baseline level as the animal's head turns away from the preferred direction (Taube et al., 1990). It has been proposed that the head-direction cells may form a ring network that allows an activity bump to be self-sustained by attractor dynamics, and the peak position of the activity bump is updated by self-motion information and calibrated by learned landmarks (Skaggs et al., 1995; Redish et al., 1996; Zhang, 1996). Multiple versions of the ring network have been studied for the head-direction cells (Goodridge and Touretzky, 2000; Arleo and Gerstner, 2001; Sharp et al., 2001; Stringer et al., 2002; Xie et al., 2002; Song and Wang, 2005) as well as for a variety of applications beyond the original head-direction system (Ben-Yishai et al., 1995; Pouget et al., 1998; Hahnloser et al., 2000; Kakaria and de Bivort, 2017; Zhang et al., 2019). Besides head-direction cells, attractor networks have been used as a general theoretical framework for modeling other types of spatial cells in the hippocampus and related systems (Knierim and Zhang, 2012).

**Figure 1:**
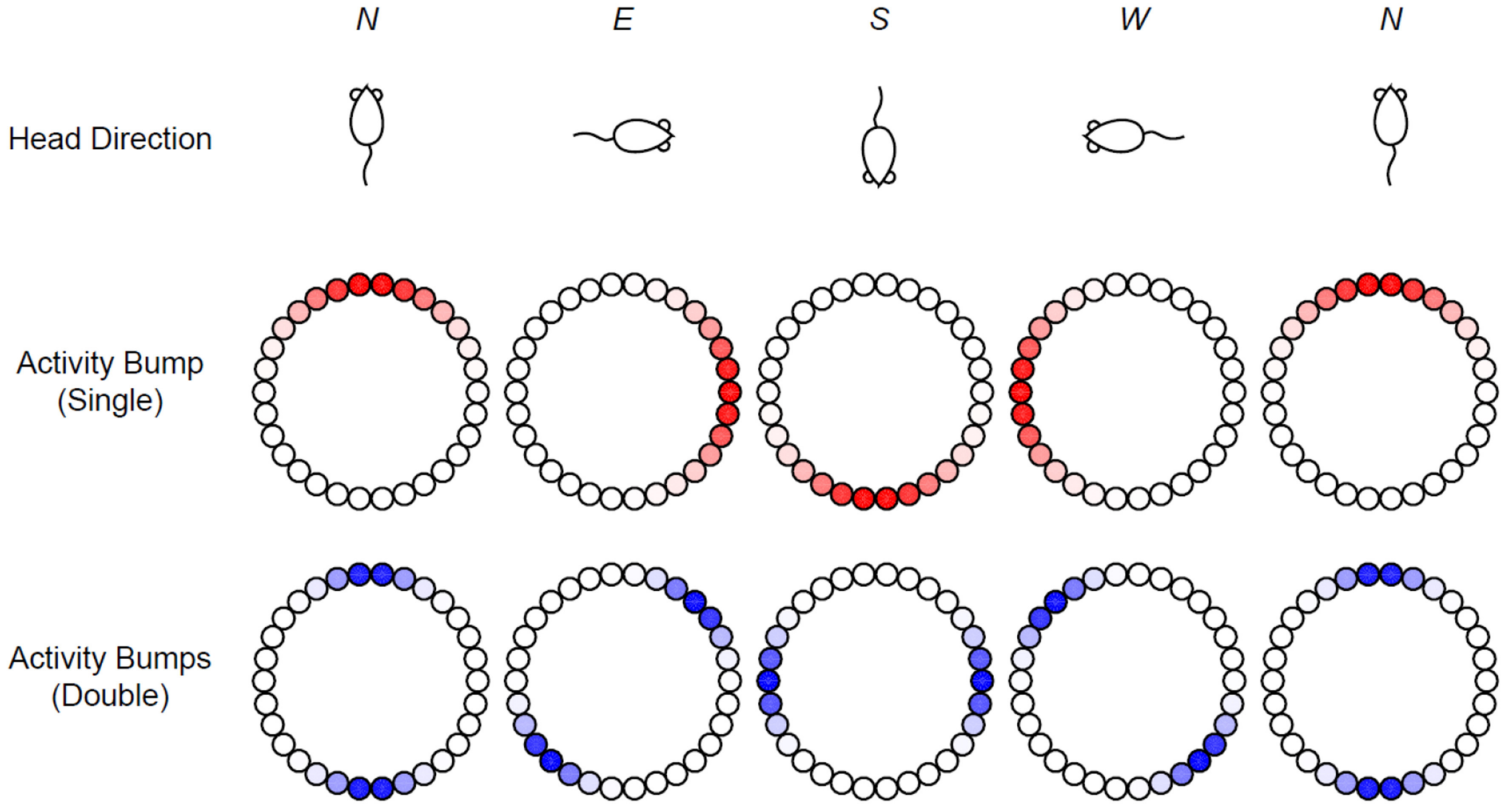
Head-direction ring network. In the classic view, the head direction of a rat **(Top)** is represented by the peak location of the activity bump (**Middle**: red shades) in a ring of head-direction cells. An alternative possibility is a ring network that allows two stable activity bumps that rotate at half of the speed **(Bottom)**.

The equilibrium state of the head-direction ring network is often visualized as a single bump of activity whose peak position corresponds to the animal's current heading direction (Figure 1, top and middle rows). While this picture is compelling and highly intuitive, it is not the only theoretical possibility for explaining the experimental data. For instance, imagine that the ring network can sustain two activity bumps instead of one (Figure 1, bottom row), then if one records from an individual cell in the ring, one would still find a head-direction cell with a perfectly normal, single-peaked tuning curve, assuming that the activity bumps now rotates at half of the speed as the single activity bump. Indeed, if we focus on a single cell corresponding to north, we see that in both situations, the cell fires at maximal rate only when the animal is facing north (*N*).

Despite the functional equivalence, the structures of the two ring networks are different. More specifically, unlike the standard single-peaked network, the double-peaked network has strong connections between cells in opposing directions although they are not as strong as the connections between neighboring cells. In the consideration above, we assume that the rotation speed is halved in the double-peaked network. If the rotation speed is kept the same, then the network should generate tuning curves with two peaks that are 180° apart. In fact, double-peaked head-direction tuning curves have been reported in the retrosplenial cortex (Jacob et al., 2017), although the phenomenon could be attributed to a single preferred direction switching back and forth in time rather than implying a truly double-peaked activity pattern (Page and Jeffery, 2018). The consideration above can be generalized readily to activity patterns with three or more peaks. The possible existence of multi-peaked activities in the head-direction system together with their potential functional significance has motivated us to examine the equilibrium states in the ring network model in greater depth.

This paper is aimed at a thorough analysis of the equilibrium states in the ring network, with a focus on the exact conditions for the existence of activity patterns with multiple peaks. We will use the simple continuous ring network to simplify the mathematical analysis. The rotational symmetry of the system allows Fourier analysis to be used effectively. We strive to derive the exact analytical conditions whenever possible, and the analytical treatments are complemented by systematic numerical simulations. Once the exact expressions of all different kinds of equilibrium states are obtained, we will employ small perturbations and eigen-analysis of the linearized system to determine the stabilities of these equilibrium states. We will examine the dependence of various equilibrium states on the network parameters and summarize the results by the phase diagrams. Our analysis may provide a necessary step for extending the application and analysis of the ring network beyond the classic single-peaked condition.

## 2. Materials and Methods

We consider a continuous formulation of the head-direction system which has a continuous ring structure (Zhang, 1996). Such continuous formulation has a long history in neural modeling (Wilson and Cowan, 1972; Amari, 1977; Bressloff, 2012). The standard simplified time evolution continuous dynamics is governed by the equation

(1) $$\tau \frac{\partial u(\theta ,t)}{\partial t}=-u(\theta ,t)+w(\theta ,t)*g(u(\theta ,t))+I(\theta ,t),\;\theta \in [0,2\pi ),$$

where the convolution is defined by

(2) $$w(\theta ,t)*g(u(\theta ,t))=\frac{1}{2\pi }\int_{0}^{2\pi }w(\theta -\varphi ,t)g(u(\varphi ,t))d\varphi .$$

In this system, *u*(θ, *t*) represents the state of voltage of a unit with θ as its preferred direction, *w*(θ − φ, *t*) represents the synaptic weight between units with θ − φ being the difference of their preferred directions, *g*(*u*) is a monotone increasing and sigmoid gain function, *I*(θ, *t*) represents external inputs, τ is a time constant, θ is head-direction, and variable *t* is time. So the whole head-direction system is given by

(3) $$\begin{array}{l} \tau \frac{\partial u(\theta ,t)}{\partial t}=-u(\theta ,t)+\frac{1}{2\pi }\int_{0}^{2\pi }w(\theta -\varphi ,t)g(u(\varphi ,t))d\varphi \\ \;+I(\theta ,t),\;\theta \in [0,2\pi ). \end{array}$$

## 3. Results

### 3.1. General Properties of Head-Direction Ring Network

#### 3.1.1. Boundedness of Solutions

According to the form of Equation (3), we multiply integrating factor $e^{\frac{t}{\tau }}$ of both sides of this equation. It is easy to get the solution of Equation (3) as follows:

(4) $$\begin{array}{l} u(\theta ,t)=\frac{u(\theta ,0)}{e^{\frac{t}{\tau }}}+\frac{\int_{0}^{t}e^{\frac{s}{\tau }}\left(\int_{0}^{2\pi }w(\theta -\varphi ,s)g(u(\varphi ,s))d\varphi \right)ds}{2\pi \tau e^{\frac{t}{\tau }}}\\ \;+\frac{\int_{0}^{t}e^{\frac{s}{\tau }}I(\theta ,s)ds}{\tau e^{\frac{t}{\tau }}}. \end{array}$$

Obviously, as *t* → +∞ the first term $u\left(\theta ,0\right)/e^{\frac{t}{\tau }}$ tends to zero. If *w*(θ, *t*) and *g*(*u*) are bounded function, then the second term is bounded. This is because:

(5) $$\begin{array}{l} \left|\int_{0}^{2\pi }w(\theta -\varphi ,s)g(u(\varphi ,s))d\varphi \right|\\ \;\leq \int_{0}^{2\pi }|w(\theta -\varphi ,s)g(u(\varphi ,s))|d\varphi \leq 2\pi \text{ M}, \end{array}$$

where M is the maximum value of the integrand. At the same time we have:

(6) $$\begin{array}{l} \left|\frac{\int_{0}^{t}e^{\frac{s}{\tau }}\left(\int_{0}^{2\pi }w(\theta -\varphi ,s)g(u(\varphi ,s))d\varphi \right)ds}{2\pi \tau e^{\frac{t}{\tau }}}\right|\\ \;\leq \frac{M\int_{0}^{t}e^{\frac{s}{\tau }}ds}{\tau e^{\frac{t}{\tau }}}\leq M,(t\to +\infty ). \end{array}$$

So when *t* → +∞ the second term of solutions (4) is bounded. In general the external inputs *I*(θ, *t*) is also bounded, thus we know that the third term of solutions (4) is bounded, too.

Therefore if synaptic weight *w*(θ, *t*), gain function *g*(*u*) and external inputs *I*(θ, *t*) are bounded, then all solutions of system (3) are tending to a bounded domain rather than wandering in the whole space. Namely as variable *t* tends to infinity, the average net state *u*(θ, *t*) for each head-direction cell is bounded and changes in a bounded domain. Meanwhile we find that if there is no external input (i.e., *I*(θ, *t*) = 0), then |*u*(θ, *t*)| is less than the maximum of the product of |*w*(θ, *t*)| and |*g*(*u*(θ, *t*))| as *t* → +∞. So the output of system (3) is under control by synaptic weight and gain function.

#### 3.1.2. The Form of Solutions

The structure of head-direction model is a homogeneous and continuous ring network, so the synaptic weight *w*(θ, *t*) and external input *I*(θ, *t*) are periodic with period 2π. We have the Fourier series expansions of *w*(θ, *t*) and *I*(θ, *t*) as follows:

(7) $$w(\theta ,t)=\sum_{n=-\infty }^{+\infty }w_{n}(t)e^{in\theta },\;I(\theta ,t)=\sum_{n=-\infty }^{+\infty }I_{n}(t)e^{in\theta }.$$

According to the form of solutions (4), we know *u*(θ, *t*) and *g*(*u*(θ, *t*)) are also periodic with period 2π on θ. So the Fourier series expansions of *u*(θ, *t*) and *g*(*u*(θ, *t*)) are given as follows:

(8) $$u(\theta ,t)=\sum_{n=-\infty }^{+\infty }u_{n}(t)e^{in\theta },\;g(u(\theta ,t))=\sum_{n=-\infty }^{+\infty }g_{n}(t)e^{in\theta }.$$

According to the convolution theorem, we know that if *g*(*u*(θ, *t*)) and *w*(θ, *t*) are in *L*^1^([−π, π]), then the Fourier series coefficients of 2π-periodic convolution of *w*(θ) and *g*(*u*(θ)) are given by:

(9) $${\left[w(\theta ,t)*g(u(\theta ,t))\right]}_{n}(t)=w_{n}(t)\cdot g_{n}(t),n=0,\pm 1,\pm 2,\cdots .$$

Therefore the Fourier series coefficients of system (3) have the following relationships:

(10) $$\tau \frac{du_{n}(t)}{dt}=-u_{n}(t)+w_{n}(t)\cdot g_{n}(t)+I_{n}(t),n=0,\pm 1,\pm 2,\cdots .$$

Notice that when external input *I*(θ, *t*) is a constant and the Fourier series coefficients of synaptic weight *w*(θ, *t*) just have finite term, i.e.,

(11) $$w(\theta ,t)=\sum_{n=-m}^{+m}w_{n}(t)e^{in\theta },$$

then when |*n*| > *m* the Fourier series coefficients of solution *u*(θ, *t*) is:

(12) $$\tau \frac{du_{n}(t)}{dt}=-u_{n}(t),$$

i.e.,

(13) $$u_{n}(t)=u_{n}(0)e^{-\frac{t}{\tau }}.$$

Obviously the Fourier series coefficients of solution *u*(θ, *t*) will reduce to zero (*t* → +∞) when |*n*| > *m*. When the synaptic weight *w*(θ, *t*) and sigmoid function *g*(*u*) are chosen in some special forms, we can derive the form and properties of the solutions, especially the equilibrium solutions.

#### 3.1.3. The Equilibrium Solutions

Until now, as far as we know the general solutions of the ring attractor network (3) can not be solved, but we can get special solutions, such as equilibrium solutions. Once the equilibrium states are determined, we can further obtain the properties of other solutions near the equilibrium solutions by local stability analysis. Sometimes we even determine the tendency of all solutions in the solution space.

Base on the definition of an equilibrium solution, we let

(14) $$\frac{\partial u(\theta ,t)}{\partial t}=0.$$

This equation shows that the equilibrium solutions is independent of time *t*. In other words, the activity *u*(θ, *t*) does not change with time. In the head-direction neural network, different equilibrium solutions are related to different equilibrium states. We write *u*(θ, *t*) = *u*(θ) which depends only on variable θ. An equilibrium solution satisfies:

(15) $$u(\theta )=\frac{1}{2\pi }\int_{0}^{2\pi }w(\theta -\varphi ,t)g(u(\varphi ))d\varphi +I(\theta ,t).$$

Here we assume *I*(θ, *t*) represents a fixed external input current (i.e., *I*(θ, *t*) = *I*), so the system has equilibrium solutions if and only if the synaptic weight *w*(θ, *t*) is also independent of time *t*. That means *w*(θ, *t*) = *w*(θ) and the equilibrium solution satisfies

(16) $$u(\theta )=\frac{1}{2\pi }\int_{0}^{2\pi }w(\theta -\varphi )g(u(\varphi ))d\varphi +I.$$

As mentioned in section 3.1.2 we know that *w*(θ, *t*), *u*(θ, *t*) and *I*(θ, *t*) are periodic with period 2π on θ. According to Equation (16) we conclude that the equilibrium solution *u*(θ) is always rotation-invariant. It means that if *u*(θ) is an equilibrium solution of system (3), then *u*(θ − θ_0_) is also an equilibrium solution. Therefore from the viewpoint of symmetry, we show that every head-direction cell in the ring network has similar equilibrium states. A mathematical verification of this conclusion is in the next paragraph.

Because *u*(θ) is an equilibrium solution which satisfies (Equation 16) for any θ_0_, we have:

(17) $$u(\theta -\theta_{0})=\frac{1}{2\pi }\int_{0}^{2\pi }w(\theta -\theta_{0}-\varphi )g(u(\varphi ))d\varphi +I.$$

Set φ = ϕ − θ_0_ and plug this relation into the integration, then we get:

(18) $$\begin{array}{l} u(\theta -\theta_{0})\\ =\frac{1}{2\pi }\int_{\theta_{0}}^{2\pi +\theta_{0}}w(\theta -\theta_{0}-(\phi -\theta_{0}))g(u(\phi -\theta_{0}))d(\phi -\theta_{0})+I. \end{array}$$

That is,

(19) $$u(\theta -\theta_{0})=\frac{1}{2\pi }\int_{0}^{2\pi }w(\theta -\phi )g(u(\phi -\theta_{0}))d\phi +I.$$

Thus *u*(θ − θ_0_) also is an equilibrium solution. According to this property there is no need to consider any shift of head direction, and we only need to focus on the mathematical forms all the equilibrium solutions.

Since constant *I* can be absorbed into the constant term of *w*(θ), we will set *I* = 0 in the following analysis. Now we choose *w*(θ) as a Fourier series with finite terms:

(20) $$\begin{array}{l} w(\theta )=a_{0}+a_{1}\text{ cos }\theta +b_{1}\text{ sin }\theta +a_{2}\text{ cos }2\theta \\ \;+b_{2}\text{ sin }2\theta +\cdots +a_{n}\text{ cos }n\theta +b_{n}\text{ sin }n\theta , \end{array}$$

and then an equilibrium solution satisfies

(21) $$\begin{array}{l} u(\theta )=\frac{1}{2\pi }\int_{0}^{2\pi }(a_{0}+a_{1}\text{ cos}(\theta -\varphi )+a_{2}\text{ cos }2(\theta -\varphi )\\ \;+\cdots +a_{n}\text{ cos }n(\theta -\varphi ))g(u(\varphi ))d\varphi \\ \;+\frac{1}{2\pi }\int_{0}^{2\pi }(b_{1}\text{ sin}(\theta -\varphi )+b_{2}\text{ sin }2(\theta -\varphi )\\ \;+\cdots +b_{n}\text{ sin }n(\theta -\varphi ))g(u(\varphi ))d\varphi . \end{array}$$

Since

(22) $$\begin{array}{l} \frac{1}{2\pi }(\int_{0}^{2\pi }a_{n}\text{ cos }n(\theta -\varphi )g(u(\varphi ))d\varphi \\ \;+\int_{0}^{2\pi }b_{n}\text{ sin }n(\theta -\varphi )g(u(\varphi ))d\varphi )=A_{n}\text{ cos }n\theta \\ \;+B_{n}\text{ sin }n\theta , \end{array}$$

where

(23) $$\begin{array}{l} A_{n}=\frac{1}{2\pi }\left(a_{n}\int_{0}^{2\pi }\text{cos }n\varphi g(u(\varphi ))d\varphi -b_{n}\int_{0}^{2\pi }\text{sin }n\varphi g(u(\varphi ))d\varphi \right),\\ B_{n}=\frac{1}{2\pi }\left(a_{n}\int_{0}^{2\pi }\text{sin }n\varphi g(u(\varphi ))d\varphi +b_{n}\int_{0}^{2\pi }\text{cos }n\varphi g(u(\varphi ))d\varphi \right), \end{array}$$

the equilibrium solution *u*(θ) has a similar form as *w*(θ), namely,

(24) $$\begin{array}{l} u(\theta )=A+A_{1}\text{ cos }\theta +B_{1}\text{ sin }\theta +A_{2}\text{ cos }2\theta +B_{2}\text{ sin }2\theta \\ \;+\cdots +A_{n}\text{ cos }\theta +B_{n}\text{ sin }\theta . \end{array}$$

Therefore we find that the form of the equilibrium states of the ring network depends heavily on the form of the synaptic weights.

#### 3.1.4. Lyapunov Function

In this section we consider the stability of system (14) by constructing a continuous version of a Lyapunov function for symmetric networks (Cohen and Grossberg, 1983; Hopfield, 1984). The Lyapunov function or energy function is as follows:

(25) $$\begin{array}{l} E=\int_{0}^{2\pi }\int_{0}^{g(u(\theta ,t))}(g^{-1}(V)-I)dVd\theta \\ \;-\frac{1}{4\pi }\int_{0}^{2\pi }\int_{0}^{2\pi }w(\theta -\varphi )g(u(\varphi ,t))g(u(\theta ,t))d\varphi d\theta . \end{array}$$

Obviously, the energy function is bounded and the time derivative of Equation (25) is:

(26) $$\begin{array}{l} dE/dt=\int_{0}^{2\pi }(g^{-1}(g(u(\theta ,t)))-I)\frac{dg(u(\theta ,t))}{dt}d\theta \\ \;-\frac{1}{4\pi }\int_{0}^{2\pi }\int_{0}^{2\pi }w(\theta -\varphi ,t)(g(u(\varphi ,t))\frac{dg(u(\theta ,t))}{dt}\\ \;+g(u(\theta ,t))\frac{dg(u(\varphi ,t))}{dt})d\varphi d\theta . \end{array}$$

We find if *w*(θ) is even, i.e., *w*(θ − φ) = *w*(φ − θ), then

(27) $$\begin{array}{l} \int_{0}^{2\pi }\int_{0}^{2\pi }w(\theta -\varphi ,t)g(u(\varphi ,t))\frac{dg(u(\theta ,t))}{dt}d\varphi d\theta \\ \;=\int_{0}^{2\pi }\int_{0}^{2\pi }w(\theta -\varphi ,t)g(u(\theta ,t))\frac{dg(u(\varphi ,t))}{dt}d\varphi d\theta . \end{array}$$

Thus its time derivative becomes:

(28) dE/dt=∫02π(u(θ,t)−I)dg(u(θ,t))dtdθ             −12π∫02π∫02πw(θ−φ,t)g(u(φ,t))dg(u(θ,t)dtdφdθ             =∫02π[u(θ,t)−I−12π∫02πw(θ             −φ,t)g(u(φ,t))dφ]dg(u(θ,t))dtdθ             =−τ∫02πdg−1(g(u(θ,t)))dg(u(θ,t))·(dg(u(θ,t))dt)d2θ

Since *V* = *g*(*u*) is a monotonically increasing gain function, its inverse function *u* = *g*^−1^(*V*) is also a monotonically increasing function, i.e., $\frac{dg^{-1}\left(V\right)}{dV}>0$. Since the time parameter τ > 0, we obtain $\frac{dE}{dt}\leq 0$, where $\frac{dE}{dt}=0$ if and only if *u*(θ, *t*) = *u*(θ) which is an equilibrium state.

Therefore when the synaptic weight *w*(θ) is even, the gain function *g*(*u*) is monotonically increasing, and the synaptic weight and the gain function are all bounded, all solutions of system (3) are convergent to the corresponding equilibrium states as *t* → +∞. If the system has one, and only one, equilibrium solution, then this equilibrium solution must have global stability. That means all flows of system (3) converge to this stable state no matter what the initial state is.

### 3.2. An Example of Head-Direction Ring Network

Based on the above analysis, in order to get all equilibrium solutions we just need to pay attention to the Fourier form of the synaptic weight. Here we choose the synaptic weight as *w*(θ) = *a*+*b* cos θ + *c* cos 2θ which only has three terms. Of course, the conclusions and methods can be extended to more general cases as long as the synaptic weight has finite terms.

According to the analysis in section 3.1.3, we know that the equilibrium solutions of system have the following form:

(29) $$u(\theta )=a_{0}+b_{1}\text{ cos }\theta +b_{2}\text{ sin }\theta +c_{1}\text{ cos }2\theta +c_{2}\text{ sin }2\theta ,$$

where,

(30) $$\begin{array}{l} a_{0}=a\int_{0}^{2\pi }g(u(\varphi ))d\varphi ,\\ b_{1}=b\int_{0}^{2\pi }\text{cos }\varphi \;g(u(\varphi ))d\varphi ,\;b_{2}=b\int_{0}^{2\pi }\text{sin }\varphi \;g(u(\varphi ))d\varphi ,\\ c_{1}=c\int_{0}^{2\pi }\text{cos }2\varphi \;g(u(\varphi ))d\varphi ,\;c_{2}=c\int_{0}^{2\pi }\text{sin }2\varphi \;g(u(\varphi ))d\varphi . \end{array}$$

Once synaptic weight is chosen, we can determine the form of equilibrium solutions. According to the solution (29), the equilibrium solution *u*(θ) has a similar form as synaptic weight *w*(θ). Therefore if the synaptic weight has no more than two peaks, then all equilibrium solutions have no more than two peaks. That is, an equilibrium of the system may be a flat solution, a single-peaked solution, or a double-peaked solution, and on the basis of relationship (30) all equilibrium solutions are dependent on control parameters *a*, *b* and *c*. In fact we can obtain all the equilibrium solutions when the sigmoid function *g*(*u*) is also chosen.

The gain function is often described by the sigmoid:

(31) $$g(u)=\frac{1}{1+e^{-k(u-u_{0})}},$$

where *k* > 0 is the gain and *u*_0_ is the threshold. For convenience in this paper we set the threshold *u*_0_ = 0. When the gain *k* is larger enough or as *k* → +∞, the form of the gain function converges to the Heaviside step function, and the derivative of the gain function reduces to the Dirac δ function.

Here we choose *I*(θ, *t*) = 0, *g*(*u*) =H(*u*) =Heaviside(*u*), and *w*(θ) = *a* + *b* cos θ + *c* cos 2θ. Now the ring network becomes:

(32) $$\begin{array}{l} \tau \frac{\partial u(\theta ,t)}{\partial t}=-u(\theta ,t)+\frac{1}{2\pi }\int_{0}^{2\pi }(a+b\text{ cos}(\theta -\varphi )\\ \;+c\text{ cos }2(\theta -\varphi ))\text{ H}(u(\varphi ,t))d\varphi . \end{array}$$

The equilibrium solution equation becomes:

(33) $$u(\theta )=\frac{1}{2\pi }\int_{0}^{2\pi }\left(a+b\text{ cos}(\theta -\varphi )+c\text{ cos }2(\theta -\varphi )\right)\text{H}(u(\varphi ))d\varphi .$$

**Figure 2:**
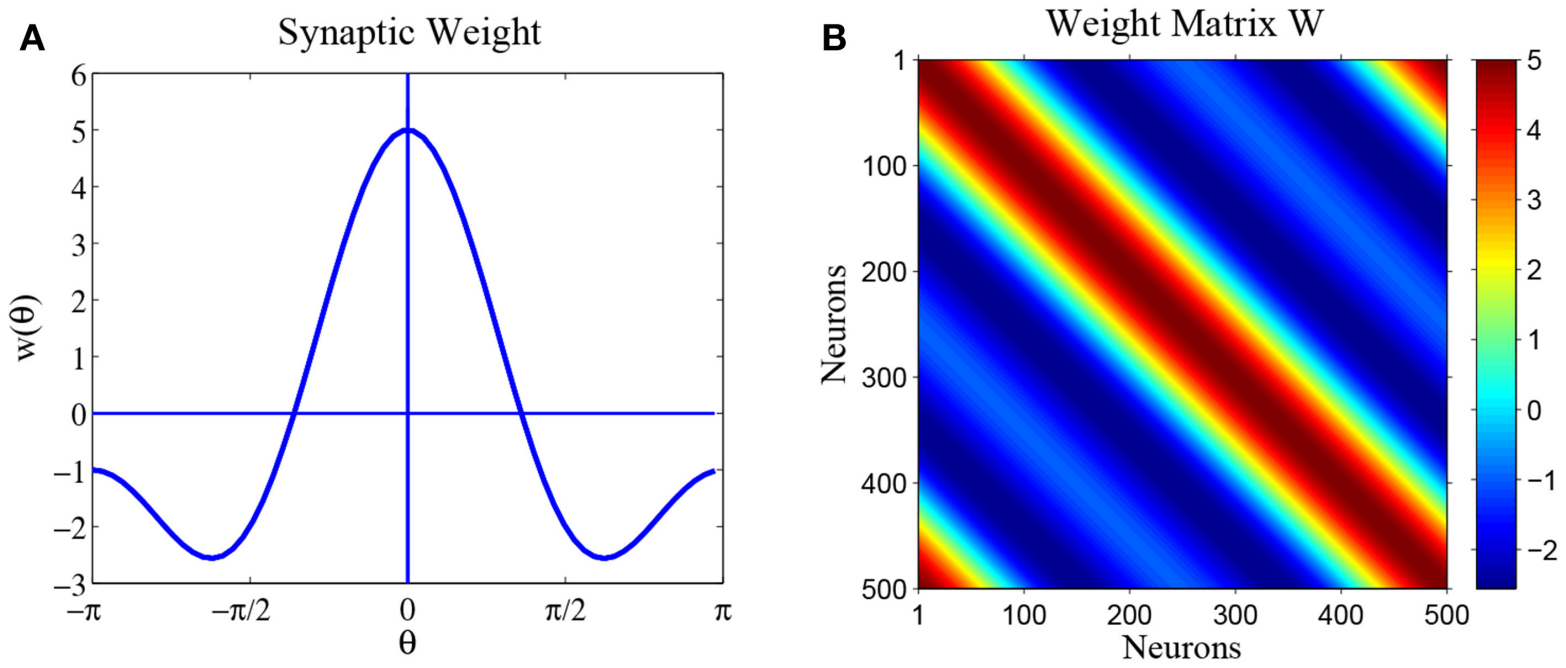
The synaptic weights for parameters *a* = 0, *b* = 3 and *c* = 2. **(A)** Diagram of weight profile *w*(θ) = *a* + *b* cos θ + *c* cos 2θ. **(B)** The synaptic weight matrix of the ring network.

Parameter *a* only moves the equilibrium state *u*(θ) up and down, without changing its shape. So we set parameter *a* = 0 in this paper. Figure 2A shows synaptic weight function *w*(θ) and Figure 2B shows the weight matrix of the ring network, with parameters *a* = 0, *b* = 3 and *c* = 2.

#### 3.2.1. All Possible Equilibrium States

The general form of the equilibrium solutions in system (32) is:

(34) $$u(\theta )=a_{0}+b_{1}\text{ cos }\theta +b_{2}\text{ sin }\theta +c_{1}\text{ cos }2\theta +c_{2}\text{ sin }2\theta .$$

Using basic trigonometric formula, we rewrite this form as:

(35) $$u(\theta )=A+B\text{ cos}(\theta -\theta_{1})+C\text{ cos }2(\theta -\theta_{2}),$$

where *A* = *a*_0_, $B=\sqrt{b_{1}^{2}+b_{2}^{2}}$, $C=\sqrt{c_{1}^{2}+c_{2}^{2}}$, $\theta_{1}=\arctan\frac{b_{2}}{b_{1}}$ and $2\theta_{2}=\arctan\frac{c_{2}}{c_{1}}$. As mentioned in section 3.1.3 for any θ_0_ if *u*(θ) is a solution of system (32), then *u*(θ − θ_0_) is also a solution. So we should pay attention only to the form of the equilibrium solutions and ignore the influence of the phase-shift θ_0_. Meanwhile according to Equation (35) we know that any equilibrium solution is a linear combination of functions cos(θ − θ_1_) and cos 2(θ − θ_2_). Next we will discuss all possible linear combinations of these three terms and solve for the exact equilibrium states respectively.

**Case 1:** *u*(θ) = *A*.

The first situation is that the equilibrium solution is a constant. If *u*(θ) = *A* is a solution of system (32), then

$$u(\theta )=\frac{\text{H}(A)}{2\pi }\int_{0}^{2\pi }(a+b\text{ cos}(\theta -\varphi )+c\text{ cos }2(\theta -\varphi ))d\varphi ,$$

i.e.,

(36) u(θ)=aH(A).

Since *a* = 0, the constant $A=a\int_{0}^{2\pi }g\left(u\left(\varphi \right)\right)d\varphi$ is always equal to zero. In other words, we have *u*(θ) = 0 as the only constant solution of the system. The general form of non-zero equilibrium solution for head-direction neural network (32) is:

(37) $$u(\theta )=B\text{ cos}(\theta -\theta_{1})+C\text{ cos }2(\theta -\theta_{2}),$$

where *B* and *C* are unknown constants and θ_1_ and θ_2_ are unknown angles.

**Case 2:** *u*(θ) = *B* cos(θ − θ_0_), *B* > 0.

Due to rotation-invariance, we just need to find one solution of this form *u*(θ) = *B* cos θ(*B* > 0). Plugging *u*(θ) = *B* cos θ(*B* > 0) into (Equation 33) we have the following equality:

$$u(\theta )=\frac{1}{2\pi }\int_{0}^{2\pi }(b\text{ cos}(\theta -\varphi )+c\text{ cos }2(\theta -\varphi ))\text{H}(B\text{ cos }\varphi )d\varphi$$

i.e.,

(38) $$u(\theta )=\frac{1}{2\pi }\int_{-\frac{\pi }{2}}^{\frac{\pi }{2}}(b\text{ cos}(\theta -\varphi )+c\text{ cos }2(\theta -\varphi ))d\varphi =\frac{b}{\pi }\text{ cos }\theta .$$

**Figure 3:**
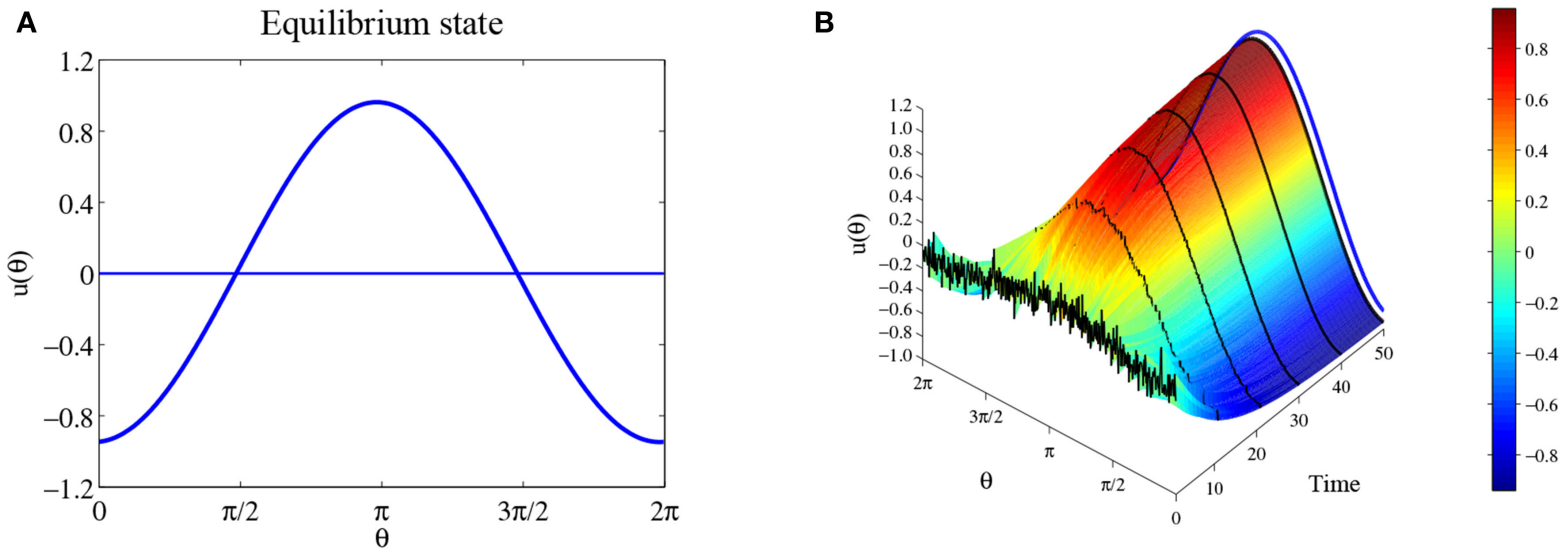
Equilibrium state for parameters *a* = 0, *b* = 3, and *c* = 2. **(A)** The equilibrium solution $u\left(\theta \right)=\frac{b}{\pi }\text{cos}\left(\theta -\pi \right)$. **(B)** The time evolution when initial state is *u*_0_ is $\frac{0.1b}{\pi }\text{cos}\left(\theta -\pi \right)$ with additional small perturbations, where the blue curve is the equilibrium solution $u\left(\theta \right)=\frac{b}{\pi }\text{cos}\left(\theta -\pi \right)$. Parameters: number of head-direction cells *N* = 500, τ = 1, time step Δ*t* = 0.1, and maximum steps 500.

So if *b* > 0, then $u\left(\theta \right)=\frac{b}{\pi }\text{cos}\left(\theta -\theta_{0}\right)$ is the equilibrium solution for any θ_0_; If *b* < 0, then there is no equilibrium solution like this form. For parameters *a* = 0, *b* = 3 and *c* = 2, we show the solution $u\left(\theta \right)=\frac{b}{\pi }\text{cos}\left(\theta -\pi \right)$ in Figure 3A. Figure 3B shows numerical simulation with initial state *u*_0_ is $0.1\frac{b}{\pi }\text{cos }\left(\theta -\pi \right)$ with additional small perturbations, and as *t* → +∞, the state approaches this equilibrium solution, suggesting that the equilibrium solution $u\left(\theta \right)=\frac{b}{\pi }\text{cos}\left(\theta -\theta_{0}\right)$ is likely to be stable. We will return this stability problem in section 3.2.2.

**Case 3:** *u*(θ) = *C* cos 2(θ − θ_0_), *C* > 0.

Similar to case 2, we just need to find one special equilibrium solution *u*(θ) = *C* cos 2θ and its coefficient *C* > 0. After that we can obtain all the equilibrium solutions with the form *u*(θ) = *C* cos 2(θ − θ_0_)(*C* > 0). Plugging *u*(θ) = *C* cos 2θ into (Equation 33) we find the following equality:

(39) $$\begin{array}{l} u(\theta )=\frac{1}{2\pi }\int_{0}^{2\pi }(b\text{ cos}(\theta -\varphi )+c\text{ cos }2(\theta -\varphi ))\text{H}(C\text{ cos }2\varphi )d\varphi \\ \;=\frac{1}{2\pi }\int_{\frac{-\pi }{4}}^{\frac{\pi }{4}}(b\text{ cos}(\theta -\varphi )+c\text{ cos }2(\theta -\varphi ))d\varphi \\ \;+\frac{1}{2\pi }\int_{\frac{3\pi }{4}}^{\frac{5\pi }{4}}(b\text{ cos}(\theta -\varphi )+c\text{ cos }2(\theta -\varphi ))d\varphi \\ \;=\frac{c}{\pi }\text{ cos }2\theta . \end{array}$$

**Figure 4:**
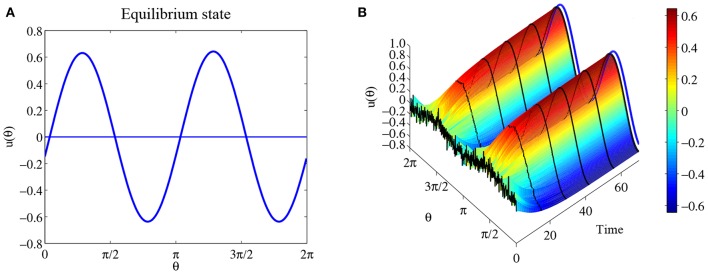
Another equilibrium state for the same parameters as Figure 3. **(A)** The equilibrium solution $u\left(\theta \right)=\frac{c}{\pi }\text{cos}\left(\theta -\frac{\pi }{2}\right)$. **(B)** The time evolution when the initial state *u*_0_ is $0.1\frac{c}{\pi }\cos2(\theta -\frac{\pi }{2}))$ with additional small perturbations. The blue curve is the equilibrium solution $u\left(\theta \right)=\frac{c}{\pi }\text{cos}\left(\theta -\frac{\pi }{2}\right)$.

So we have the following conclusion. If *c* > 0, then $u\left(\theta \right)=\frac{c}{\pi }\text{cos }2\left(\theta -\theta_{0}\right)$ is the equilibrium solution for system (32) for any θ_0_; If *c* < 0, then there is no equilibrium solution like this form. Figure 4A shows solution $u\left(\theta \right)=\frac{c}{\pi }\text{cos}\left(\theta -\frac{\pi }{2}\right)$ for parameters *a* = 0, *b* = 3 and *c* = 2. Figure 4B shows the time evolution with initial state $u_{0}=0.1\frac{c}{\pi }\text{cos }2(\theta -\frac{\pi }{2}))$ with additional small perturbation. The result indicates that this equilibrium solution is probably stable.

**Case 4:** *u*(θ) = *B* cos(θ − θ_1_) + *C* cos 2(θ − θ_2_), *B* > 0 and *C* > 0.

When the equilibrium solutions have form *B* cos(θ − θ_1_) + *C* cos 2(θ − θ_2_), we change the form of equilibrium solution as follows:

(40) $$B\text{ cos}(\theta -\theta_{1})+C\text{ cos }2(\theta -\theta_{1}-(\theta_{2}-\theta_{1}))=B\text{ cos }\phi +C\text{ cos }2(\phi -\phi_{0}),$$

where ϕ = θ − θ_1_ and ϕ_0_ = θ_2_ − θ_1_. Due to the property of equilibrium solutions we just need to find the basic equilibrium solutions

(41) $$u(\phi )=B\text{ cos }\phi +C\text{ cos }2(\phi -\phi_{0}).$$

**Figure 5:**
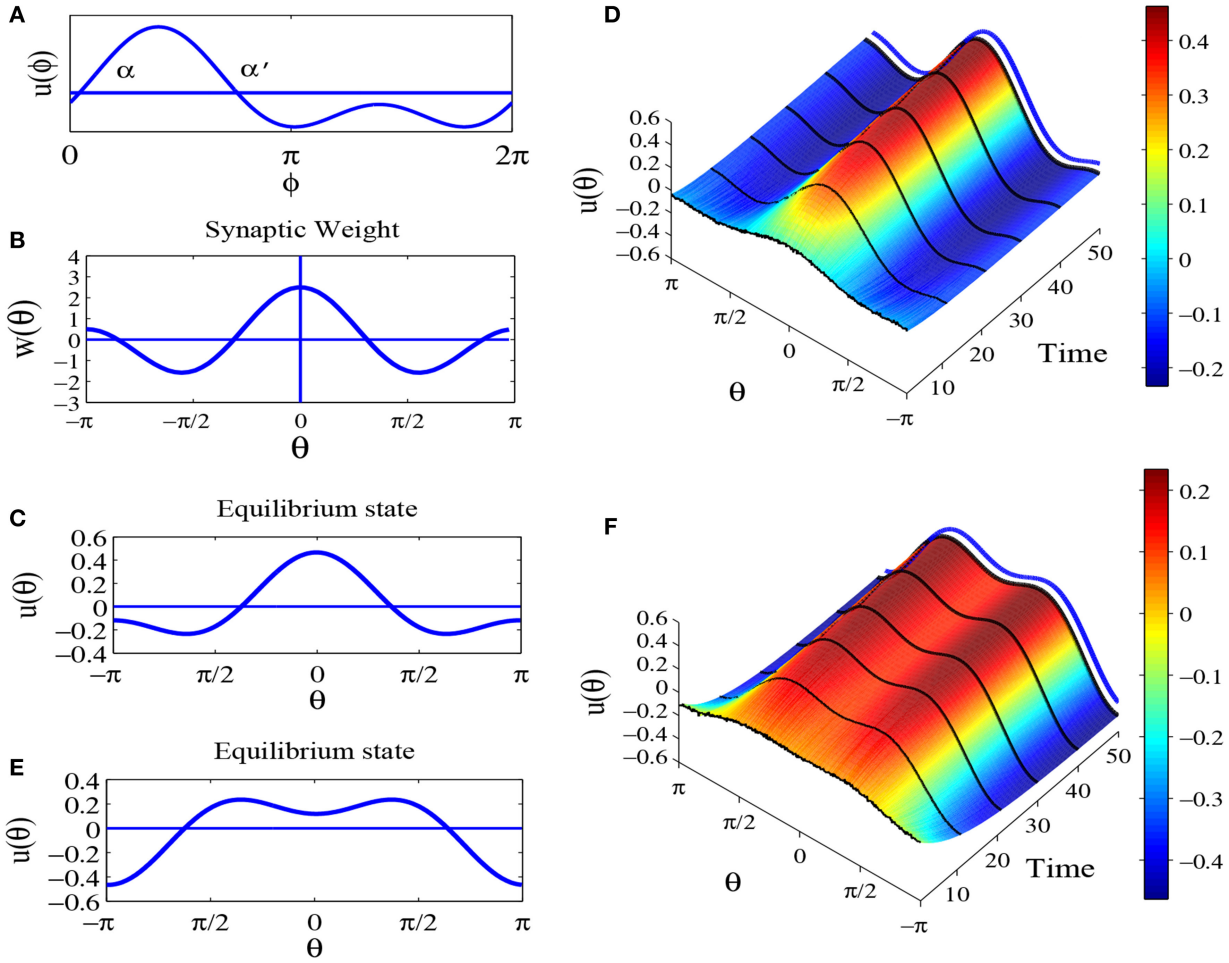
Equilibrium states for parameters *a* = 0, *b* = 1, and *c* = 1.5, with other parameters the same as Figure 3. **(A)** A sketch of *u*(θ) = *B* cos ϕ + *C* cos 2(ϕ − ϕ_0_), *B* ≥ *C* > 0. **(B)** The profile of synaptic weight function *w*(θ) = *a* + *b* cos θ + *c* cos 2θ. **(C)** The equilibrium solution $u_{1}\left(\theta \right)=\frac{b}{\pi }\sqrt{\frac{c+b}{2c}}\text{cos }\theta +$ $\frac{\sqrt{c^{2}-b^{2}}}{2\pi }\text{cos }2\theta$. **(D)** The time evolution for initial state *u*_0_ = 0.1*u*_1_(θ) with additional small perturbations. The blue lines indicates the equilibrium solution. **(E)** The equilibrium solution $u_{2}\left(\theta \right)=\frac{b}{\pi }\sqrt{\frac{c+b}{2c}}\text{cos }\theta -\frac{\sqrt{c^{2}-b^{2}}}{2\pi }\text{cos }2\theta$. **(F)** The time evolution for initial state *u*_0_ = 0.1*u*_2_(θ) with additional small perturbations. The blue lines indicates the equilibrium solution.

By numerical simulation we find that the phase diagram of *u*(ϕ) has two situations as parameters *B*, *C* and ϕ_0_ are changing. One situation is there is only one positive domain above ϕ-axis in period 2π, another situation is there are two positive domains above ϕ-axis in period 2π. Because positive domain decides the value of Heaviside function, we have to discuss these two cases respectively. In this section we mainly consider the first situation in which *u*(ϕ) just has one positive domain above ϕ-axis in period 2π. that is shown in Figure 5A.

As a equilibrium solution, we plug *u*(ϕ) = *B* cos ϕ + *C* cos 2(ϕ − ϕ_0_) into Equation (33) to obtain

(42) $$\begin{array}{l} u(\phi )=B\text{ cos }\phi +C\text{ cos }2(\phi -\phi_{0})=\frac{1}{2\pi }\int_{0}^{2\pi }(b\text{ cos}(\phi -\varphi )\\ \;+c\text{ cos }2(\phi -\varphi ))H(B\text{ cos }\phi +C\text{ cos }2(\phi -\phi_{0}))d\varphi \\ \;=\frac{1}{2\pi }\int_{\alpha }^{\alpha^{\prime }}(b\text{ cos}(\phi -\varphi )+c\text{ cos }2(\phi -\varphi ))d\varphi , \end{array}$$

i.e.,

(43) $$\begin{array}{l} u\left(\phi \right)=\frac{b}{\pi }\text{sin }\frac{\alpha^{\prime }-\alpha }{2}\text{cos }\left(\phi -\frac{\alpha^{\prime }+\alpha }{2}\right)\\ \;+\frac{c}{2\pi }\text{sin }\left(\alpha^{\prime }-\alpha \right)\text{ cos }2\left(\phi -\frac{\alpha^{\prime }+\alpha }{2}\right). \end{array}$$

For any value ϕ (Equation 43) must hold, which means that the corresponding coefficients must be equal. So we get $\sin\left(\frac{\alpha^{\prime }+\alpha }{2}\right)=0$, i.e., sin(α′ + α) = 0 and sin 2ϕ_0_ = 0. This result shows that in this case if the system has an equilibrium solution, then its form must be *u*(ϕ) = *B* cos ϕ ± *C* cos 2ϕ. And the parameters *B* and *C* must satisfy the following relationship:

(44) $$\begin{array}{l} B\text{ cos}\phi \pm C\text{ cos }2\phi =\frac{b}{\pi }\text{sin }\alpha \text{ cos }\phi +\frac{c}{2\pi }\text{sin }2\alpha \text{ cos }2\phi . \end{array}$$

Comparing the corresponding coefficients we have $B=\frac{b}{\pi }\sin\alpha$ and $\pm C=\frac{c}{2\pi }\sin2\alpha$. Since α is a root of equation *u*(ϕ) = 0, next we should solve equation *u*(ϕ) = 0. We find sin α = 0, cos α = 0 and $\text{cos }2\alpha =-\frac{b}{c}$. Because *B* > 0 and *C* > 0, we keep $\text{cos }2\alpha =-\frac{b}{c}$ and reject the others. If $\text{cos }2\alpha =-\frac{b}{c}$ is a valid root, we have $|\frac{b}{c}|<1$, i.e., |*b*| < |*c*|. So we obtain $\sin\alpha =\sqrt{\frac{c+b}{2c}}$ and $\text{cos }\alpha =\pm \sqrt{\frac{c-b}{2c}}$. At the same time we have $B=\frac{b}{\pi }\sqrt{\frac{c+b}{2c}}$ and $C=\frac{c}{\pi }\sqrt{\frac{c^{2}-b^{2}}{4c^{2}}}$. For the equations to hold there is only one positive domain in period 2π, we have *B* ≥ *C* > 0, i.e., $\frac{b}{\pi }\sqrt{\frac{c+b}{2c}}\geq \frac{c}{\pi }\sqrt{\frac{c^{2}-b^{2}}{4c^{2}}}>0$, and the parameter sb and c must satisfy 2*b* ≥ *c* > 0. Comprehensively, in the first situation if the system has the equilibrium solution *u*(ϕ) = *B* cos ϕ ± *C* cos 2ϕ, then parameters *b* and *c* must satisfy the condition 0 < *b* < *c* ≤ 2*b*. So we have the following conclusion: If 0 < *b* < *c* ≤ 2*b*, then for any θ_0_ the system has an equilibrium solution of the form:

(45) $$\begin{array}{l} u\left(\theta \right)=\frac{b}{\pi }\sqrt{\frac{c+b}{2c}}\text{ cos }\left(\theta -\theta_{0}\right)\pm \frac{\sqrt{c^{2}-b^{2}}}{2\pi }\text{cos }2\left(\theta -\theta_{0}\right). \end{array}$$

For parameters *a* = 0, *b* = 1 and *c* = 1.5, the profile of the synaptic weight is shown in Figure 5B. By numerical simulation we find two of this kind solutions as shown in Figures 5C,E, while Figures 5D,F are time evolution with initial state *u*_0_ = 0.1*u*(θ) with additional small perturbations. The results suggest that this type of solutions may be stable too.

**Figure 6:**
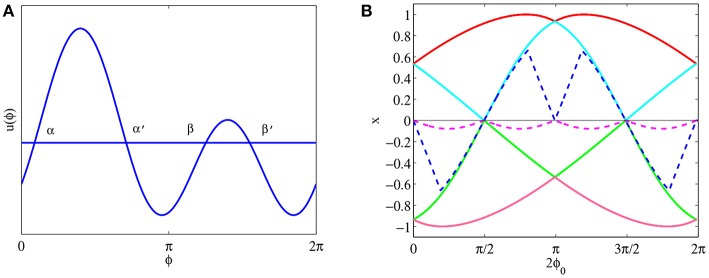
**(A)** A sketch of *u*(θ) = *B* cos ϕ + *C* cos 2(ϕ − ϕ_0_), 0 < *B* < *C*. **(B)** Four solid lines represent the four roots of Equation (49), i.e., cos α, cos α′, cos β and cos β′, while two dash lines represent all possibilities of cos − cos α′ + cos β − cos β′. Parameter *k* = 0.8.

Now we consider the second situation two domains above ϕ-axis in period 2π as shown in Figure 6A. As above analysis we know the basic form of this kind equilibrium solution is *u*(ϕ) = *B* cos ϕ + *C* cos 2(ϕ − ϕ_0_). Plug *u*(ϕ) into the equilibrium solution Equation (33), we get

(46) $$\begin{array}{l} u(\phi )=B\text{ cos }\phi +C\text{ cos }2(\phi -\phi_{0})=\frac{1}{2\pi }\int_{0}^{2\pi }(b\text{ cos}(\phi -\varphi )\\ \;+c\text{ cos }2(\phi -\varphi ))H(B\text{ cos }\phi +C\text{ cos }2(\phi -\phi_{0}))d\varphi \\ \;=\frac{1}{2\pi }\int_{\alpha }^{\alpha^{\prime }}(b\text{ cos}(\phi -\varphi )+c\text{ cos }2(\phi -\varphi ))d\varphi \\ \;+\frac{1}{2\pi }\int_{\beta }^{\beta^{\prime }}(b\text{ cos}(\phi -\varphi )+c\text{ cos }2(\phi -\varphi ))d\varphi \\ \;=\frac{b}{2\pi }(\text{ cos }\alpha -\text{ cos }\alpha^{\prime }+\text{ cos }\beta -\text{ cos }\beta^{\prime })\text{ sin }\phi \\ \;-\frac{b}{2\pi }(\text{ sin }\alpha -\text{ sin }\alpha^{\prime }+\text{ sin }\beta -\text{ sin }\beta^{\prime })\text{ cos }\phi \\ \;+\frac{c}{4\pi }(\text{ cos }2\alpha -\text{ cos }2\alpha^{\prime }+\text{ cos }2\beta -\text{ cos }2\beta^{\prime })\text{ sin }2\phi \\ \;-\frac{c}{4\pi }(\text{ sin }2\alpha -\text{ sin }2\alpha^{\prime }+\text{ sin }2\beta -\text{ sin }2\beta^{\prime })\text{ cos }2\phi . \end{array}$$

Compare the corresponding relationship we have cos α − cos α′ + cos β − cos β′ = 0, where α, α′, β and β′ are four roots of equation *u*(ϕ) = 0. That means we have to solve equation *u*(ϕ) = *B* cos ϕ + *C* cos 2(ϕ − ϕ_0_) = 0. Using trigonometric formulas to expand *u*(ϕ) = 0 the equation *u*(ϕ) = 0 becomes:

(47) $$\begin{array}{l} B\text{ cos }\phi +C\left(2{\text{ cos }}^{2}\phi -1\right)\text{ cos }2\phi_{0}+2C\text{ sin }\phi \text{ cos }\phi \text{ sin }2\phi_{0}=0. \end{array}$$

For convenience let *x* = cos ϕ and $k=\frac{B}{C}$ we square both side of the Equation (47), then the Equation (47) becomes

(48) $$\begin{array}{l} {\left(kx+\left(2x^{2}-1\right)\text{ cos }2\phi_{0}\right)}^{2}=4\left(1-x^{2}\right)x^{2}\left(1-\text{ cos }2\phi_{0}^{2}\right), \end{array}$$

i.e.,

(49) $$\begin{array}{l} 4x^{4}+4k\text{ cos }2\phi_{0}x^{3}+\left(k^{2}-4\right)x^{2}-2k\text{ cos }2\phi_{0}x+{\text{cos }}^{2}2\phi_{0}=0. \end{array}$$

According to the four roots of Equation (49), we can find that if and only if 2ϕ_0_ is equals to $0,\frac{\pi }{2},\pi$ and $\frac{3\pi }{2}$, then the roots of Equation (47) hold the corresponding relation cos α − cos α′ + cos β − cos β′ = 0. This result can be seen in Figure 6B. Four solid lines represent four roots of Equation (49), and two dash lines are all possible combination of cos α − cos α′ + cos β − cos β′. According to Figure 6B we find that if and only if 2ϕ_0_ equals 0, $\frac{\pi }{2}$, π and $\frac{3\pi }{2}$, then four roots of Equation (47) satisfy cos α − cos α′ + cos β − cos β′ = 0, where *k* = 0.8. By using numerical simulation we find when *k* is selected other positive number, although the amount of wing flexing for all lines are changing, the positions of all points of intersection on 2ϕ_0_-axis are immovability. And the change of picture is successive. That means if and only if 2ϕ_0_ is equals to $0,\frac{\pi }{2},\pi$ and $\frac{3\pi }{2}$, then the system may be exist equilibrium solutions which have two domains above ϕ-axis. Let us consider these four situations one by one.

**(1)** 2ϕ_0_ = 0.

If 2ϕ_0_ = 0, then system (32)may be have one kind of equilibrium solutions which like *u*(ϕ) = *B* cos ϕ + *C* cos 2ϕ which has two positive domain in period 2π. In this case the roots of *u*(θ) = 0 are −α, α, β and 2π − β. So plug *u*(ϕ) = *B* cos ϕ + *C* cos 2ϕ into (Equation 33), and then we obtain the relationship as follow

(50) $$\begin{array}{l} u\left(\phi \right)=B\text{ cos }\phi +C\text{ cos }2\phi =\frac{b}{\pi }\left(\text{sin }\alpha -\text{ sin }\beta \right)\text{ cos }\phi \\ \;+\frac{c}{2\pi }\left(\text{sin }2\alpha -\text{ sin }2\beta \right)\text{ cos }2\phi . \end{array}$$

Since −α, α, β and 2π − β are four roots of equation *u*(θ) = 0, thus we should get the following the following equalities:

(51) $$\left\{ \begin{array}{l} \frac{b}{\pi }(\text{sin }\alpha -\text{ sin }\beta )\text{ cos }\alpha +\frac{c}{2\pi }(\text{sin }2\alpha -\text{ sin }2\beta )\text{ cos }2\alpha =0,\\ \frac{b}{\pi }(\text{sin }\alpha -\text{ sin }\beta )\text{ cos }\beta +\frac{c}{2\pi }(\text{sin }2\alpha -\text{ sin }2\beta )\text{ cos }2\beta =0. \end{array}\right.$$

Let the first equation of (51) minus the second equation of (51), we have

(52) $$\begin{array}{l} \frac{b}{\pi }\left(\text{sin }\alpha -\text{ sin }\beta \right)\left(\text{cos }\alpha -\text{ cos }\beta \right)\\ \;+\frac{c}{2\pi }\left(\text{sin }2\alpha -\text{ sin }2\beta \right)\left(\text{cos }2\beta -\text{ cos }2\beta \right)=0. \end{array}$$

Since cos 2β − cos 2β = 2(sin β − sin α)(sin β + sin α) and sin α ≠ sin β, thus the above equality becomes:

(53) $$\begin{array}{l} b\left(\text{cos }\alpha -\text{ cos }\beta \right)-c\left(2\text{ sin }\alpha \text{ cos }\alpha -2\text{ sin }\beta \text{ cos }\beta \right)\left(\text{sin }\beta +\text{ sin }\alpha \right)=0. \end{array}$$

For −α, α, β and 2π − β are four roots of equation *u*(ϕ) = *B* cos ϕ + *C* cos 2ϕ = 0, we get:

(54) $$\begin{array}{l} 2C{\text{ cos }}^{2}\phi +B\text{ cos }\phi -C=0. \end{array}$$

So we have $\text{cos }\alpha \cdot \text{cos }\beta =-\frac{1}{2}$. Plug $\text{cos }\beta =-\frac{1}{2\text{cos }\alpha }$ into (Equation 53) and compute cos α. In order to keep meaningful during computational process we set 0 < *b* < 2*c* and $\frac{b}{c}\neq \frac{1}{2}$, then we have $\text{cos }\alpha =\sqrt{\frac{s-\sqrt{s^{2}-1}}{2}}$, where $s=\frac{2-\frac{b}{c}+\sqrt{\frac{2b}{c}}}{2}$. Therefore we have the following conclusion:

If 0 < *b* < 2*c* and $\frac{b}{c}\neq \frac{1}{2}$, then for any θ_0_ the system has equilibrium solutions which are

(55) $$\begin{array}{l} u(\theta )=\frac{b}{\pi }(\sqrt{1-m^{2}}-\frac{\sqrt{4m^{2}-1}}{2m})\text{cos}(\theta -\theta_{0})+\frac{c}{\pi }(m\sqrt{1-m^{2}}\\ \;+\frac{\sqrt{4m^{2}-1}}{4m^{2}})\text{cos }2(\theta -\theta_{0}), \end{array}$$

where

(56) $$\begin{array}{l} m=\text{ cos }\alpha =\frac{\sqrt{2-\frac{b}{c}+\sqrt{\frac{2b}{c}}-\sqrt{\frac{b^{2}}{c^{2}}-\frac{2b}{c}-\frac{2b}{c}\sqrt{\frac{2b}{c}}+4\sqrt{\frac{2b}{c}}}}}{2}. \end{array}$$

**(2)** 2ϕ_0_ = π.

When 2ϕ_0_ = π the analyzing and calculating process is the same as 2ϕ_0_ = 0. Because of this we omit the complex calculation and present the conclusion directly.

If 0 < *b* < 2*c* and $\frac{b}{c}\neq \frac{1}{2}$, then for any θ_0_ the system has equilibrium solutions which are

(57) $$\begin{array}{l} u\left(\theta \right)=\frac{b}{\pi }\left(\frac{\sqrt{4m^{2}-1}}{2m}-\sqrt{1-m^{2}}\right)\text{ cos }\left(\theta -\theta_{0}\right)\\ \;-\frac{c}{\pi }\left(\frac{\sqrt{4m^{2}-1}}{4m^{2}}+m\sqrt{1-m^{2}}\right)\text{ cos }2\left(\theta -\theta_{0}\right), \end{array}$$

where

(58) $$\begin{array}{l} m=\text{ cos }\alpha =\frac{\sqrt{2-\frac{b}{c}+\sqrt{2\frac{b}{c}}+\sqrt{\frac{b^{2}}{c^{2}}-2\frac{b}{c}-2\frac{b}{c}\sqrt{2\frac{b}{c}}+4\sqrt{2\frac{b}{c}}}}}{2}. \end{array}$$

**Figure 7:**
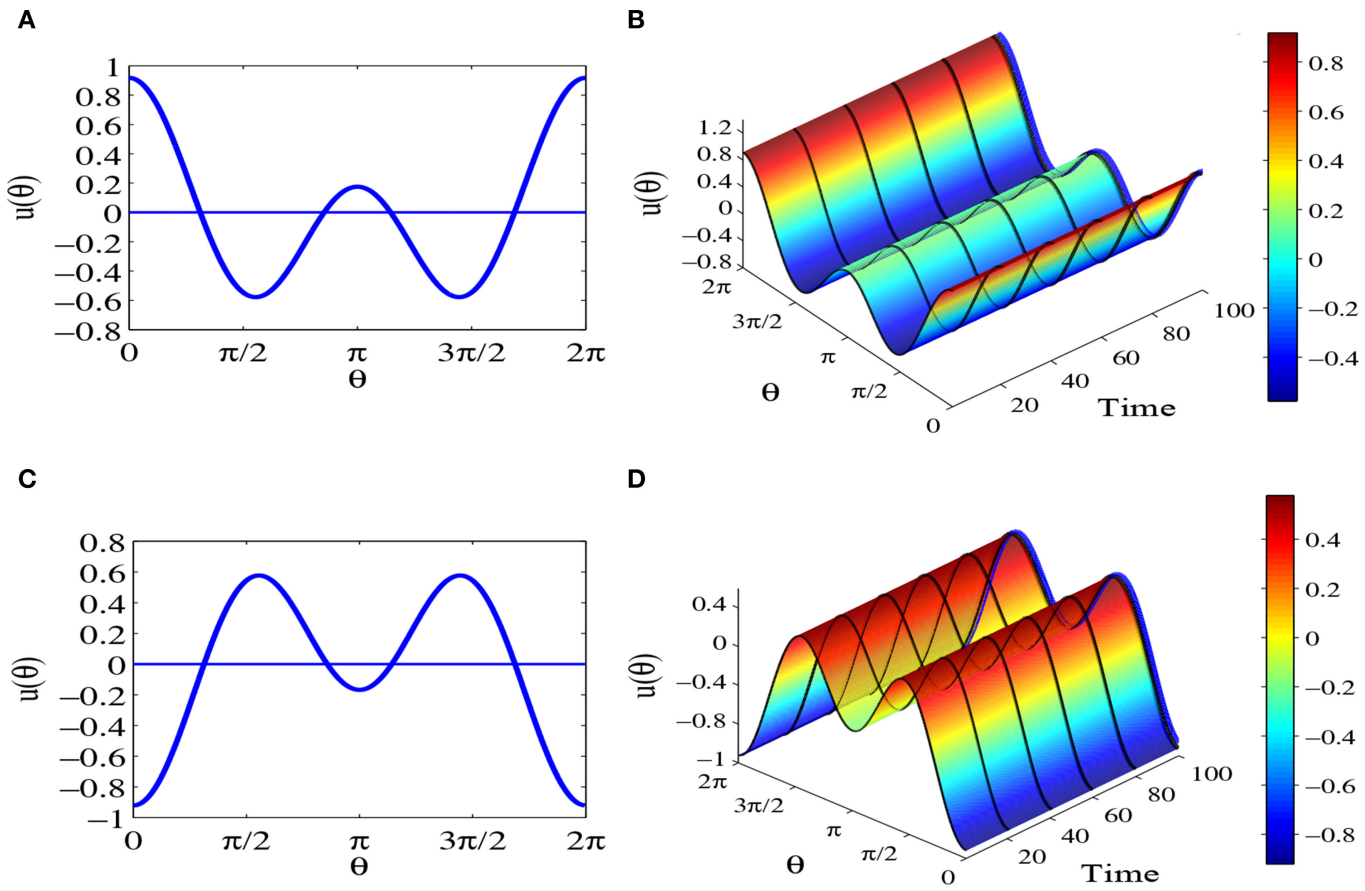
Equilibrium states for parameters *a* = 0, *b* = 3 and *c* = 2, which satisfy 0 < *b* < 2*c* and $\frac{b}{c}\neq \frac{1}{2}$. The network contains *N* = 1000 head-direction cells, τ = 1 and time step Δ*t* = 0.1. **(A)** The equilibrium solution (55). **(B)** The time evolution of the equilibrium solution (55), where blue line represents the equilibrium solution (55). **(C)** The equilibrium solution (57). **(D)** The time evolution of equilibrium solution (57), where blue line represents the equilibrium solution (57).

When parameters set *a* = 0, *b* = 3 and *c* = 2 and 0 < *b* < 2*c*, Figures 7A,C show the equilibrium solution (55) and equilibrium solution (57). Meanwhile we can see the time evolution of solution (55) and solution (57) from Figures 7B,D. Obviously, these two kinds of equilibrium solutions keep immovability when *t* tends to infinity.

**(3)** $2\phi_{0}=\frac{\pi }{2}$.

If $2\phi_{0}=\frac{\pi }{2}$, then the equilibrium solutions of system (32) like *u*(ϕ) = *B* cos ϕ + *C* sin 2ϕ. In this case the four roots of *u*(ϕ) = 0 are −α, $\frac{\pi }{2}$, π + α and $\frac{3\pi }{2}$. As one kind of equilibrium solution we Plug *u*(ϕ) = *B* cos ϕ + *C* sin 2ϕ into equation and then we obtain the relation as follow:

(59) $$\begin{array}{l} u\left(\theta \right)=B\text{ cos }\theta +C\text{ sin }2\theta =\frac{b}{\pi }\text{sin }\alpha \text{ cos }\theta +\frac{c}{\pi }{\text{cos}}^{2}\alpha \text{ sin }2\theta . \end{array}$$

Since −α is root of equation *u*(θ) = 0, then we have:

(60) $$\begin{array}{l} u\left(-\alpha \right)=\frac{b}{\pi }\text{sin }\alpha \text{ cos }\alpha -\frac{c}{\pi }{\text{cos}}^{2\;}\alpha \text{ sin }2\alpha =0 \end{array}$$

i.e.,

(61) $$\begin{array}{l} b\text{ sin }\alpha \text{ cos }\alpha -2c{\text{ cos}}^{3}\alpha \text{ sin }\alpha =0. \end{array}$$

Solve above equation and get the roots cos α = 0, sin α = 0 and ${\text{cos}}^{2\;}\alpha =\frac{b}{2c}$. For *B* > 0 and *C* > 0 we choose ${\text{cos}}^{2\;}\alpha =\frac{b}{2c}$, other roots are given up. Of course, the parameter must meet $0<\frac{b}{2c}<1$, i.e., 0 < *b* < 2*c*. Therefore we have $\text{sin}\alpha =\sqrt{\frac{2c-b}{2c}}$. Now we get another form equilibrium solution which is

(62) $$\begin{array}{l} u\left(\theta \right)=\frac{b}{\pi }\sqrt{\frac{2c-b}{2c}}\text{ cos }\theta +\frac{b}{2\pi }\text{sin }2\theta . \end{array}$$

In a words, if 0 < *b* < 2*c*, then for any θ_0_ the system has equilibrium solutions which are:

(63) $$\begin{array}{l} u\left(\theta \right)=\frac{b}{\pi }\sqrt{\frac{2c-b}{2c}}\text{cos}\left(\theta -\theta_{0}\right)+\frac{b}{2\pi }\text{sin }2\left(\theta -\theta_{0}\right). \end{array}$$

**Figure 8:**
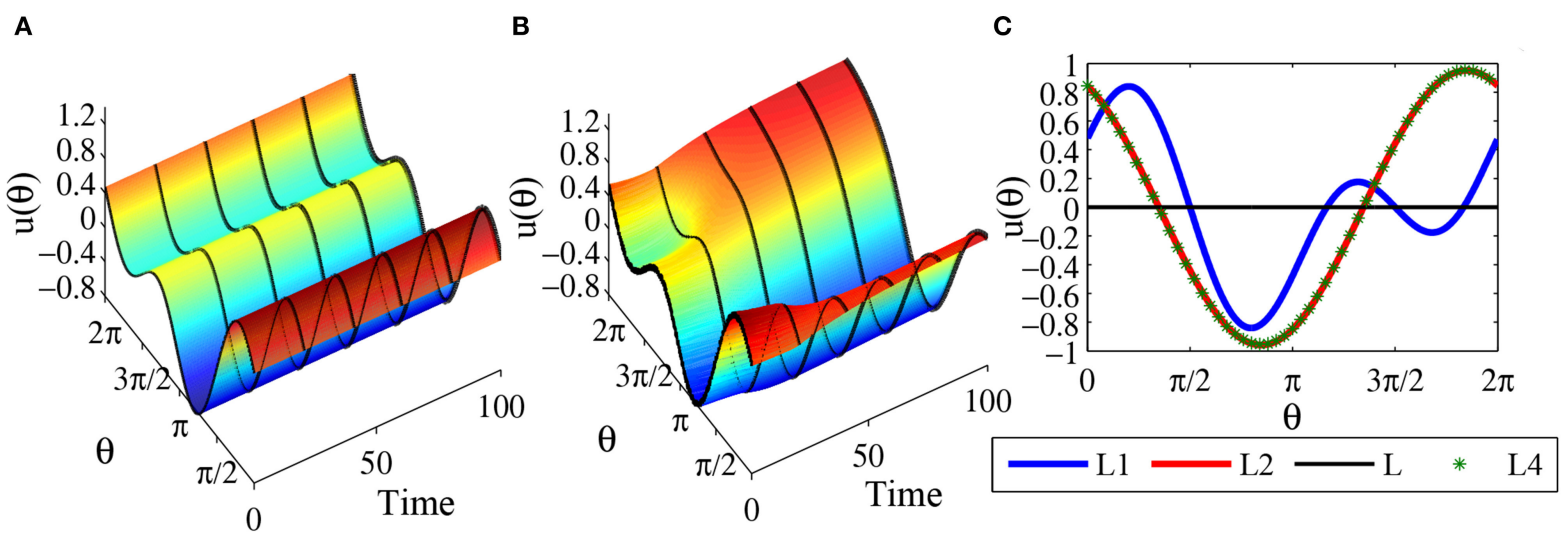
Equilibrium states for parameters *a* = 0, *b* = 3, and *c* = 2, which satisfy 0 < *b* < 2*c* and $\frac{b}{c}\neq \frac{1}{2}$. The network contains *N* = 1000 head-direction cells, τ = 1 and time step Δ*t* = 0.1. **(A)** The time evolution of equilibrium solution (63) conforms that the state is stationary. **(B)** The time evolution of equilibrium solution (63) with disturbances in the initial state indicates that the equilibrium is unstable. **(C)** Comparison chart, where the blue line represents the equilibrium solution (63), the red line represents the final state starting from (63) after 100, 000 time steps, which matches perfectly with the one-peaked stable equilibrium solution $u\left(\theta \right)=\frac{b}{\pi }\text{cos}\left(\theta -\frac{6.5\pi }{42}\right)$ as indicated by the green stars.

When parameters are chosen *a* = 0, *b* = 3 and *c* = 2, which satisfy 0 < *b* < 2*c*, you must get this equilibrium solution. Figure 8A is time evolution of equilibrium solutions (63).

**(4)** $2\phi_{0}=\frac{3\pi }{2}$.

Actually when $2\phi_{0}=\frac{3\pi }{2}$ the analysis and calculating process is in the same way as $2\phi_{0}=\frac{\pi }{2}$. Here we ignore the complex calculating process and give the follow conclusion:

If 0 < *b* < 2*c*, then for any θ_0_ the system has equilibrium solution which are

(64) $$\begin{array}{l} u\left(\theta \right)=\frac{b}{\pi }\sqrt{\frac{2c-b}{2c}}\text{cos}\left(\theta -\theta_{0}\right)-\frac{b}{2\pi }\text{sin }2\left(\theta -\theta_{0}\right). \end{array}$$

We find that this kind solution is mirror-symmetry with equilibrium solution (63). Actually in equilibrium solution (63) if we replace θ − θ_0_ with −(θ − θ_0_), then we get equilibrium solution (64). So we can image the graph of equilibrium solution (64) according to equilibrium solution (63). To our surprise, we find that equilibrium solution (63) and equilibrium solution (64) are two asymmetric equilibrium activity pattern even when the network connectivity pattern is strictly symmetric.

#### 3.2.2. The Local Stability of Equilibrium States

In order to analyze the stability of equilibrium solutions we select *n* head-direction neural cells which have the preferred direction from 0 to 2π, and they are $\frac{1\cdot 2\pi }{n}$, $\frac{2\cdot 2\pi }{n}$, …, $\frac{n\cdot 2\pi }{n}$. Therefore the head-direction neural network which contains *n* units is as follows

(65) $$\begin{array}{l} \tau \frac{\partial }{\partial t}\left(\begin{array}{c} u\left(\theta_{1},t\right)\\ u\left(\theta_{2},t\right)\\ \vdots \\ u\left(\theta_{n},t\right) \end{array}\right)=-\left(\begin{array}{c} u\left(\theta_{1},t\right)\\ u\left(\theta_{2},t\right)\\ \vdots \\ u\left(\theta_{n},t\right) \end{array}\right)\\ +\frac{1}{n}\left(\begin{array}{cccc} w\left(\theta_{1}-\theta_{1}\right) & w\left(\theta_{1}-\theta_{2}\right) & \ldots & w\left(\theta_{1}-\theta_{n}\right)\\ w\left(\theta_{2}-\theta_{1}\right) & w\left(\theta_{2}-\theta_{2}\right) & \ldots & w\left(\theta_{2}-\theta_{n}\right)\\ \vdots & \vdots & \ddots & \vdots \\ w\left(\theta_{n}-\theta_{1}\right) & w\left(\theta_{n}-\theta_{2}\right) & \ldots & w\left(\theta_{n}-\theta_{n}\right) \end{array}\right)\left(\begin{array}{c} g\left(u\left(\theta_{1},t\right)\right)\\ g\left(u\left(\theta_{2},t\right)\right)\\ \vdots \\ g\left(u\left(\theta_{n},t\right)\right) \end{array}\right). \end{array}$$

We set solution *u*(θ, *t*) = *u*(θ) + ε(θ, *t*), where *u*(θ) is a equilibrium solution and ε(θ, *t*) is a small perturbation. Use Taylor's expansion to expand *g*(*u*(θ, *t*)) at *u*(θ), we have:

(66) $$g(u(\theta ,t))=g(u(\theta )+\varepsilon (\theta ,t))=g(u(\theta ))+g^{\prime }(u(\theta ))\varepsilon (\theta ,t))+O(\varepsilon^{2}).$$

So as to further consider the perturbation equation of equilibrium solutions. We plug Taylor's expansion of *g*(*u*(θ, *t*)) into Equation (65), then we have

(67) $$\begin{array}{l} \tau \frac{\partial E}{\partial t}=-\left(U+E\right)+\frac{1}{n}W\left(G_{1}+GE\right)+O\left(E^{2}\right) \end{array}$$

where

(68) $$\begin{array}{l} E=(\begin{array}{l} \varepsilon (\theta_{1},t)\\ \varepsilon (\theta_{2},t)\\ \;\vdots \\ \varepsilon (\theta_{n},t) \end{array}),\\ W=(\begin{array}{llll} w(\theta_{1}-\theta_{1}) & w(\theta_{1}-\theta_{2}) & \ldots & w(\theta_{1}-\theta_{n})\\ w(\theta_{2}-\theta_{1}) & w(\theta_{2}-\theta_{2}) & \ldots & w(\theta_{2}-\theta_{n})\\ \vdots & \vdots & \ddots & \vdots \\ w(\theta_{n}-\theta_{1}) & w(\theta_{n}-\theta_{2}) & \ldots & w(\theta_{n}-\theta_{n}) \end{array}),\\ U=\left(\begin{array}{l} u(\theta_{1})\\ u(\theta_{2})\\ \vdots \\ u(\theta_{n}) \end{array}\right),G_{1}=\left(\begin{array}{l} g(u(\theta_{1}))\\ g(u(\theta_{2}))\\ \vdots \\ g(u(\theta_{n})) \end{array}\right),\\ G=(\begin{array}{llll} g^{\prime }(u(\theta_{1})) & \;0 & \ldots & 0\\ 0 & g^{\prime }(u(\theta_{2})) & \ldots & 0\\ \vdots & \vdots & \ddots & \vdots \\ 0 & 0 & \ldots & g^{\prime }(u(\theta_{n})) \end{array}), \end{array}$$

Since *u*(θ) is the equilibrium solution, we have $U=\frac{1}{n}WG_{1}$. Thus head-direction neural network (67) can be simplified as follows:

(69) $$\begin{array}{l} \tau \frac{\partial E}{\partial t}=-E+\frac{1}{n}WGE+O\left(E^{2}\right)=\left(-I+\frac{1}{n}WG\right)\cdot E+O\left(E^{2}\right), \end{array}$$

and the linear part is

(70) $$\begin{array}{l} \tau \frac{\partial E}{\partial t}=JE, \end{array}$$

where $J=-I+\frac{1}{n}WG$ is the Jacobian matrix and *I* is *n* × *n* identity matrix. The general solution to this linear ordinary differential equations is:

(71) $$\begin{array}{l} E=c_{1}e^{\lambda_{1}t}V_{1}+c_{2}e^{\lambda_{2}t}V_{2}+\cdots +c_{n}e^{\lambda_{n}t}V_{n}, \end{array}$$

where λ_1_, λ_2_, ⋯, λ_*n*_ are eigenvalues of the Jacobian matrix, *V*_1_, *V*_2_, ⋯, *V*_*n*_ are corresponding eigenvectors, and *c*_1_, *c*_2_, ⋯, *c*_*n*_ are any constant. It is easy to obtain that the stability of perturbation (Equation 69) depends on the eigenvectors of the Jacobian matrix. Since synaptic weight function *w*(θ) is even and periodic, the corresponding matrix *W* is a circulatory and symmetric matrix. According to properties of a circulatory and symmetric matrix (Gray, 2005; Tee, 2007) we can get the normalized and orthogonal eigenvectors of $\frac{1}{n}W$. They are

(72) $$\begin{array}{l} V_{j}=\frac{1}{\sqrt{n}}{\left(\rho_{j}^{0},\rho_{j}^{1},\cdots \;,\rho_{j}^{n-1}\right)}^{T},j=0,1,\cdots \;,n-1, \end{array}$$

where $\rho_{j}=\exp\left(\frac{2\pi i}{n}j\right)$ (*j* = 0, 1, ⋯, *n*−1) are the *n*-th roots of unity and *i* is the imaginary unit. The corresponding eigenvalues6 are given by

(73) $$\begin{array}{l} \lambda_{j}=\frac{1}{n}{\left(w\left(0\frac{2\pi }{n}\right)\rho_{j}^{0}+w\left(1\frac{2\pi }{n}\right)\rho_{j}^{1}+\cdots +w\left(\left(n-1\right)\frac{2\pi }{n}\right)\rho_{j}^{n-1}\right)}^{T},\\ \;j=0,1,\cdots \;,n-1. \end{array}$$

Since

(74) $$\begin{array}{l} \rho_{j}^{n-k}=\text{exp}\left(j\frac{\left(n-k\right)2\pi }{n}i\right)=\text{exp}\left(j\frac{-k2\pi }{n}i\right)=\bar{\text{exp}\left(j\frac{k2\pi }{n}i\right)}\\ \;=\bar{\rho_{j}^{k}},j=0,1,\cdots \;,n-1, \end{array}$$

thus

(75) $$\begin{array}{l} \rho_{j}^{n-k}+\rho_{j}^{k}=\bar{\rho_{j}^{k}}+\rho_{j}^{k}=2\text{ cos }\left(\frac{2k\pi }{n}j\right),j=0,1,\cdots \;,n-1. \end{array}$$

So we change the form of eigenvalues as follows:

(76) $$\begin{array}{l} {\text{λ}}_{j}=\frac{1}{n}[w(0\frac{2\pi }{n})\text{cos}(0\frac{2\pi }{n}j)+w(1\frac{2\pi }{n})\text{cos}(1\frac{2k\pi }{n}j)\\ \;+\cdots +w((n-1)\frac{2\pi }{n})\text{cos}((n-1)\frac{2\pi }{n}j)]. \end{array}$$

i.e.,

(77) $$\begin{array}{l} \lambda_{j}=\frac{1}{n}\overset{n-1}{\underset{k=0}{\sum }}w\left(k\frac{2\pi }{n}\right)\text{ cos }\left(k\frac{2\pi }{n}j\right),j=0,1,\cdots \;,n-1. \end{array}$$

As *n* tends to infinite the eigenvalues become:

(78) $$\begin{array}{l} \lambda_{j}=\underset{n\to \infty }{\text{lim}}\frac{1}{n}\overset{n-1}{\underset{k=0}{\sum }}w\left(k\frac{2\pi }{n}\right)\text{cos}\left(k\frac{2\pi }{n}j\right)=\frac{1}{2\pi }\int_{0}^{2\pi }w\left(\varphi \right)\text{cos}\left(j\varphi \right)d\varphi ,\\ \;j=0,1,\cdots \;,n-1. \end{array}$$

Because of the synaptic weight *w*(θ) = *b* cos θ + *c* cos 2θ, the eigenvalues of matrix $\frac{1}{n}W$ are:

(79) $$\begin{array}{l} \lambda_{0}=0,\lambda_{1}=\frac{b}{2},\lambda_{2}=\frac{c}{2},\lambda_{3}=0,\cdots \;,\lambda_{n-1}=0. \end{array}$$

Set *V* = (*V*_0_, *V*_1_, *V*_2_, ⋯, *V*_*n*−1_), obviously *V* is an normal orthogonal matrix and the characteristic equation becomes

(80) $$\begin{array}{l} f\left(\lambda \right)=\left|V^{T}\left(\lambda +1\right)IV-V^{T}\left(\frac{1}{n}WG\right)V\right|\\ \;=|\left(\lambda +1\right)I-\left(V^{T}\left(\frac{1}{n}W\right)V\right)\left(V^{T}GV\right)|, \end{array}$$

where

(81) $$V^{T}\frac{1}{n}WV=\left(\begin{array}{cccccc} 0 & 0 & 0 & 0 & \cdots & 0\\ 0 & \frac{b}{2} & 0 & 0 & \cdots & 0\\ 0 & 0 & \frac{c}{2} & 0 & \cdots & 0\\ 0 & 0 & 0 & 0 & \cdots & 0\\ \vdots & \vdots & \vdots & \vdots & \ddots & \vdots \\ 0 & 0 & 0 & 0 & \cdots & 0 \end{array}\right)=K.$$

We have the characteristic equation as follows

(82) $$\begin{array}{l} f\left(\lambda \right)=|\left(\lambda +1\right)I-K\left(V^{T}GV\right)|. \end{array}$$

Now we can obtain all the eigenvalues from Equation (82) when the equilibrium solution is chosen, and then we can further determine the stability of each equilibrium solution.

**Case 1:** *u*(θ) = 0.

Since *u*(θ) = 0, we have

(83) $$V^{T}GV=V^{T}\left(\begin{array}{cccc} g^{\prime }(0) & 0 & \cdots & 0\\ 0 & g^{\prime }(0) & \cdots & 0\\ \vdots & \vdots & \ddots & \vdots \\ 0 & 0 & \cdots & g^{\prime }(0) \end{array}\right)V=g^{\prime }(0)V^{T}IV=g^{\prime }(0)I.$$

Thus

(84) $$\begin{array}{l} V^{T}(\frac{1}{n}W)V\cdot V^{T}GV=g^{\prime }(0)\cdot I\cdot \left(\begin{array}{llllll} 0 & 0 & 0 & 0 & \cdots & 0\\ 0 & \frac{b}{2} & 0 & 0 & \cdots & 0\\ 0 & 0 & \frac{c}{2} & 0 & \cdots & 0\\ 0 & 0 & 0 & 0 & \cdots & 0\\ \vdots & \vdots & \vdots & \vdots & \ddots & \vdots \\ 0 & 0 & 0 & 0 & \cdots & 0 \end{array}\right)\\ 13g\;=\left(\begin{array}{llllll} 0 & 0 & 0 & 0 & \cdots & 0\\ 0 & g^{\prime }(0)\frac{b}{2} & 0 & 0 & \cdots & 0\\ 0 & 0 & g^{\prime }(0)\frac{c}{2} & 0 & \cdots & 0\\ 0 & 0 & 0 & 0 & \cdots & 0\\ \vdots & \vdots & \vdots & \vdots & \ddots & \vdots \\ 0 & 0 & 0 & 0 & \cdots & 0 \end{array}\right) \end{array}$$

So the eigenvalues of characteristic Equation (82) are

(85) $$\begin{array}{l} \lambda_{1}=-1,\lambda_{2}=-1+\frac{g^{\prime }\left(0\right)b}{2},\\ \lambda_{3}=-1+\frac{g^{\prime }\left(0\right)c}{2},\lambda_{4}=\ldots =\lambda_{n}=-1. \end{array}$$

Here *g*(*u*) is a Heaviside function and *g*′(*u*) is Dirac delta function. That means *g*′(0) = +∞. So if *b* < 0 and *c* < 0 all the eigenvalues are negative, then equilibrium solution *u*(θ) = 0 is stable. Otherwise, if *b* > 0 or *c* > 0 there is at least one eigenvalue larger than 0, then equilibrium solution *u*(θ) = 0 is unstable.

In order to discuss the stability of other equilibrium solutions, we set $\left(V^{T}\left(\frac{1}{n}W\right)V\right)\left(V^{T}GV\right)={\left(g_{i,j}\right)}_{n\times n,}$ thus the characteristic Equation (82) is given by

$$\begin{array}{l} f\left(\lambda \right)={\left(\lambda +1\right)}^{n-2}\cdot |\begin{array}{c} \lambda +1-\frac{b}{2}g_{22}\lambda +1-\frac{b}{2}g_{23}\\ \lambda +1-\frac{c}{2}g_{32}\lambda +1-\frac{c}{2}g_{33} \end{array}| \end{array}$$

i.e.,

(86) $$\begin{array}{l} f(\text{λ})={\left(\text{λ}+1\right)}^{n-2}({\left(\text{λ}+1\right)}^{2}-(\frac{b}{2}g_{22}+\frac{c}{2}g_{33})(\text{λ}+1)\\ \;+\frac{bc}{4}(g_{22}g_{33}-g_{23}g_{32})), \end{array}$$

where

(87) $$\begin{array}{l} g_{22}=\frac{1}{n}(g^{\prime }(u(\theta_{1})){\left(\rho_{1}^{0}\right)}^{2}+g^{\prime }(u(\theta_{2})){\left(\rho_{1}^{1}\right)}^{2}\\ \;+\cdots +g^{\prime }(u(\theta_{n})){\left(\rho_{1}^{n-1}\right)}^{2}),\\ g_{33}=\frac{1}{n}(g^{\prime }(u(\theta_{1})){\left(\rho_{2}^{0}\right)}^{2}+g^{\prime }(u(\theta_{2})){\left(\rho_{2}^{1}\right)}^{2}\\ \;+\cdots +g^{\prime }(u(\theta_{n})){\left(\rho_{2}^{n-1}\right)}^{2}),\\ g_{32}=g_{32}=\frac{1}{n}(g^{\prime }(u(\theta_{1}))\rho_{1}^{0}\rho_{2}^{0}+g^{\prime }(u(\theta_{2}))\rho_{1}^{1}\rho_{2}^{1}\\ \;+\cdots +g^{\prime }(u(\theta_{n}))\rho_{1}^{n-1}\rho_{2}^{n-1}). \end{array}$$

As *n* approaches infinity, Equation(87) become

(88) $$\begin{array}{l} g_{22}=\frac{1}{2\pi }\int_{0}^{2\pi }g^{\prime }(u(\theta ))e^{2\theta i}d\theta ,g_{33}=\frac{1}{2\pi }\int_{0}^{2\pi }g^{\prime }(u(\theta ))e^{4\theta i}d\theta ,\\ g_{23}=g_{32}=\frac{1}{2\pi }\int_{0}^{2\pi }g^{\prime }(u(\theta ))e^{3\theta i}d\theta . \end{array}$$

So we can further determine the stability of other equilibrium solutions.

**Case 2:** $u\left(\theta \right)=\frac{b}{\pi }\text{cos }\theta ,b>0$.

When the equilibrium is $u\left(\theta \right)=\frac{b}{\pi }\text{cos }\theta \left(b>0\right)$, we have:

(89) $$\begin{array}{l} g_{22}=\frac{1}{2\pi }\left(\int_{0}^{\pi }g^{\prime }(u(\theta ))e^{2i\theta }d\theta +\int_{\pi }^{2\pi }g^{\prime }(u(\theta ))e^{2i\theta }d\theta \right)\\ \;=\frac{1}{2\pi }(\int_{\frac{b}{\pi }}^{-\frac{b}{\pi }}g^{\prime }(u)e^{2i\arccos(\frac{\pi }{b}u)}\frac{-\frac{\pi }{b}}{\sqrt{1-{\left(\frac{\pi }{b}u\right)}^{2}}}du\\ \;+\;\int_{-\frac{b}{\pi }}^{\frac{b}{\pi }}g^{\prime }(u)e^{2i(2\pi -\arccos(\frac{\pi }{b}u))}\frac{\frac{\pi }{b}}{\sqrt{1-{\left(\frac{\pi }{b}u\right)}^{2}}}du)=-\frac{1}{b}. \end{array}$$

Use the same computing method, we obtain

(90) $$g_{33}=\frac{1}{b},\;g_{32}=g_{32}=0.$$

Therefore characteristic (Equation 82) is

(91) $$f(\lambda )={\left(\lambda +1\right)}^{n-2}({\left(\lambda +1\right)}^{2}+(\frac{1}{2}-\frac{c}{2b})(\lambda +1)-\frac{c}{4b}).$$

Thus the eigenvalues are

(92) $${\text{λ}}_{1}=\cdots ={\text{λ}}_{n-2}=-1,{\text{λ}}_{n-1}=-\frac{3}{2},{\text{λ}}_{n}=-1+\frac{c}{2b}.$$

Because of *b* > 0 when parameters satisfy *c* < 2*b* all eigenvalues are negative, thus equilibrium solution $u\left(\theta \right)=\frac{b}{\pi }\text{cos }\theta \left(b>0\right)$ is stable.

**Case 3:** $u\left(\theta \right)=\frac{c}{\pi }\text{cos }2\theta ,c>0$.

When the equilibrium is $u\left(\theta \right)=\frac{c}{\pi }\text{cos }2\theta \left(c>0\right)$, by computation we get

(93) $$g_{22}=0,\;g_{33}=-\frac{1}{c},\;g_{32}=g_{32}=0.$$

Therefore the characteristic Equation (82) is

(94) $$f(\lambda )={\left(\lambda +1\right)}^{n-1}((\lambda +1)+\frac{1}{2}),$$

and the eigenvalues are:

(95) $${\text{λ}}_{1}=\cdots ={\text{λ}}_{n-1}=-1,{\text{λ}}_{n}=-\frac{3}{2}.$$

All the eigenvalues are negative, so $u\left(\theta \right)=\frac{c}{\pi }\text{cos }2\theta \left(c>0\right)$ is stable.

**Case 4:** $u\left(\theta \right)=\frac{b}{\pi }\sqrt{\frac{c+b}{2c}}\text{cos }\theta \pm \frac{\sqrt{c^{2}-b^{2}}}{2\pi }\text{cos }2\theta ,0<b<c\leq 2b$.

When the equilibrium is $u\left(\theta \right)=\frac{b}{\pi }\sqrt{\frac{c+b}{2c}}\text{cos }\theta +\frac{c}{\pi }\frac{\sqrt{c^{2}-b^{2}}}{2c}\text{cos }2\theta \left(0<b<c\leq 2b\right)$, we obtain

(96) $$\begin{array}{l} g_{22}=\frac{-2b}{2c^{2}+bc-b^{2}}\;g_{33}=\frac{4b^{2}-2c^{2}}{(2c^{2}+bc-b^{2})c},\\ \;g_{32}=g_{32}=\frac{-2(2b+c)\sqrt{\frac{c-b}{2c}}}{2c^{2}+bc-b^{2}}. \end{array}$$

Therefore characteristic equation is

(97) $$\begin{array}{l} f(\text{λ})={\left(\text{λ}+1\right)}^{n-2}({\left(\text{λ}+1\right)}^{2}+\frac{c^{2}-b^{2}}{2c^{2}+bc-b^{2}}(\text{λ}+1)\\ \;-\frac{b^{2}c^{2}+bc^{3}}{2{\left(2c^{2}+bc-b^{2}\right)}^{2}}). \end{array}$$

For convenient we set $k=\frac{c}{b}$, the Equation (97) becomes

(98) $$\begin{array}{l} f(\text{λ})={\left(\text{λ}+1\right)}^{n-2}({\left(\text{λ}+1\right)}^{2}+\frac{k^{2}-1}{2k^{2}+k-1}(\text{λ}+1)\\ \;-\frac{k^{2}+k^{3}}{2{\left(2k^{2}+k-1\right)}^{2}}). \end{array}$$

Thus the eigenvalues are

(99) $$\begin{array}{l} {\text{λ}}_{1}=\cdots ={\text{λ}}_{n-2}=-1,{\text{λ}}_{n-1,n}=-1\\ +\frac{-1+2k+k^{2}-2k^{3}\pm \sqrt{1-4k+4k^{2}+2k^{3}-7k^{4}+4k^{5}+4k^{6}}}{2(1-3k+4k^{3})}. \end{array}$$

Obviously when parameters satisfy 0 < *b* < *c* ≤ 2*b*, i.e., 1 < *k* ≤ 2, all eigenvalues are negative. So the equilibrium solution $u\left(\theta \right)=\frac{b}{\pi }\sqrt{\frac{c+b}{2c}}\text{cos }\theta +\frac{c}{\pi }\frac{\sqrt{c^{2}-b^{2}}}{2c}\text{cos }2\theta$ is stable. By the same analysis method we also get that solution $u\left(\theta \right)=\frac{b}{\pi }\sqrt{\frac{c+b}{2c}}\text{cos }\theta -\frac{c}{\pi }\frac{\sqrt{c^{2}-b^{2}}}{2c}\text{cos }2\theta$ is stable too.

According to the Equation (86), we know that at least *n*−2 eigenvalues of the four remaining equilibrium solutions are equals to −1 and at most two eigenvalues are different. So we just need to consider these different eigenvalues in each case. And the two different eigenvalues satisfy equation

$$f_{1}(\text{λ})={\left(\text{λ}+1\right)}^{2}-(\frac{b}{2}g_{22}+\frac{c}{2}g_{33})(\text{λ}+1)+\frac{bc}{4}(g_{22}g_{33}-g_{23}g_{32}),$$

thus all the eigenvalues are:

$$\begin{array}{l} {\text{λ}}_{1}=\cdot \cdot \cdot ={\text{λ}}_{n-2}=-1,\;{\text{λ}}_{n-1,n}\\ \;=-1+\frac{bg_{22}+cg_{33}\pm \sqrt{{\left(bg_{22}-cg_{33}\right)}^{2}+4bc(g_{22}g_{33}-g_{23}g_{32})}}{4}, \end{array}$$

Since *g*_23_ = *g*_32_, we have

$$\begin{array}{l} \lambda_{n-1,n}=-1+\frac{bg_{22}+cg_{33}\pm \sqrt{{\left(bg_{22}-cg_{33}\right)}^{2}+4bcg_{23}^{2}}}{4}. \end{array}$$

Since ${\left(bg_{22}-cg_{33}\right)}^{2}+4bcg_{23}^{2}>0$, we know all eigenvalues of the four remaining equilibrium solutions are real numbers and the greatest eigenvalue is

(100) $${\text{λ}}_{\max}={\text{λ}}_{n}=-1+\frac{bg_{22}+cg_{33}+\sqrt{{\left(bg_{22}-cg_{33}\right)}^{2}+4bcg_{23}^{2}}}{4},$$

where,

$$\begin{array}{l} g_{22}=\frac{1}{2\pi }\int_{0}^{2\pi }g^{\prime }(u(\theta ))e^{2\theta i}d\theta ,\;g_{33}=\frac{1}{2\pi }\int_{0}^{2\pi }g^{\prime }(u(\theta ))e^{4\theta i}d\theta ,\\ g_{23}=\frac{1}{2\pi }\int_{0}^{2\pi }g^{\prime }(u(\theta ))e^{3\theta i}d\theta . \end{array}$$

Obviously if λ_max_ < 0, all eigenvalues are negative, so that the corresponding equilibrium solution is stable. If λ_max_ > 0, at least one eigenvalue is positive, so that the corresponding equilibrium solution is unstable. Therefore putting the equilibrium solution into Equation (100) allows allows us to determine the stability of the equilibrium solution.

For example, in the equilibrium solution $u\left(\theta \right)=\frac{b}{\pi }\sqrt{\frac{2c-b}{2c}}\text{cos}\left(\theta -\theta_{0}\right)+\frac{b}{2\pi }\text{sin }2\left(\theta -\theta_{0}\right)$ (63) we set the parameters as *a* = 0, *b* = 3, *c* = 2, and θ_0_ = 0 to obtain $u\left(\theta \right)=\frac{3}{2\pi }\left(1+2\text{sin }\theta \right)\text{cos }\theta$. Plugging this equilibrium solution into (Equation 100), we find that the maximum eigenvalues of the equilibrium solutions are positive. This means that the equilibrium solution (63) are unstable. That is, adding small perturbations to the equilibrium solutions (63) will make the state moving away from the equilibrium solutions (63), as shown in Figure 8B. In fact the state approaches other stable equilibrium solution. As illustrated in Figure 8B we find that the state converges to the one-peaked stable equilibrium solution given by $u_{\theta }=\frac{3}{\pi }\text{cos}\left(\theta -\theta_{0}\right)$. This result is confirmed in Figure 8C where the blue line is the equilibrium solution (63), the red line represents the state of the system after 100, 000 time steps starting from the equilibrium solution (63) as the initial state, and the green star line is the one-peaked stable equilibrium solution $u\left(\theta \right)=\frac{3}{\pi }\text{cos}\left(\theta -\frac{6.5\pi }{42}\right)$. We can see that the system starts from an unstable equilibrium state but converges gradually to this stable equilibrium.

#### 3.2.3. Phase Diagram in the Parameter Space of the Ring Network

So far we have obtained all kinds of equilibrium solutions and determined their stability when the synaptic weight is *w*(θ) = *a*+*b* cos θ + *c* cos 2θ and sigmoid function is *g*(*u*) = Heaviside(*u*). We find that the form of these solutions are strongly dependent on weight coefficients *a*, *b* and *c*. The values of these parameters determine not only the form of the equilibrium solutions, but also their stability. Setting parameter *a* = 0, we summarize the results in section 3.2.1 with the following conclusion.

**Proposition**:

- For any *b* and *c*, system (32) has one, and only one, constant equilibrium solution *u*(θ) = 0;
- When *b* > 0, system (32) has symmetric and one-peaked equilibrium solutions $u\left(\theta \right)=\frac{b}{\pi }\text{cos}\left(\theta -\theta_{0}\right)$;
- When *c* > 0, system (32) has symmetric and two-peaked equilibrium solutions $u\left(\theta \right)=\frac{c}{\pi }\text{cos }2\left(\theta -\theta_{0}\right)$;
- When 0 < *b* < *c* < 2*b*, the system (32) has the equilibrium solutions
  ④ $$u(\theta )=\frac{b}{\pi }\sqrt{\frac{c+b}{2c}}\text{cos}(\theta -\theta_{0})\pm \frac{\sqrt{c^{2}-b^{2}}}{2\pi }\text{cos }2(\theta -\theta_{0});$$
- When 0 < *b* < 2*c*, the system (32) has four kinds of special equilibrium solutions:
  $$\begin{array}{l} u\left(\theta \right)=\frac{b}{\pi }\sqrt{\frac{2c-b}{2c}}\text{ cos }\left(\theta -\theta_{0}\right)\pm \frac{b}{2\pi }\text{sin }2\left(\theta -\theta_{0}\right) \end{array}$$

and

⑤ $$\begin{array}{l} u(\theta )=\pm \frac{b}{\pi }(\sqrt{1-m^{2}}-\frac{\sqrt{4m^{2}-1}}{2m})\text{cos}(\theta -\theta_{0})\\ \pm \frac{c}{\pi }(m\sqrt{1-m^{2}}+\frac{\sqrt{4m^{2}-1}}{4m^{2}})\text{cos }2(\theta -\theta_{0}), \end{array}$$

where

$$\begin{array}{l} m=\frac{\sqrt{2-\frac{b}{c}+\sqrt{\frac{2b}{c}}\mp \sqrt{\frac{b^{2}}{c^{2}}-\frac{2b}{c}-\frac{2b}{c}\sqrt{\frac{2b}{c}}+4\sqrt{\frac{2b}{c}}}}}{2}. \end{array}$$

**Figure 9:**
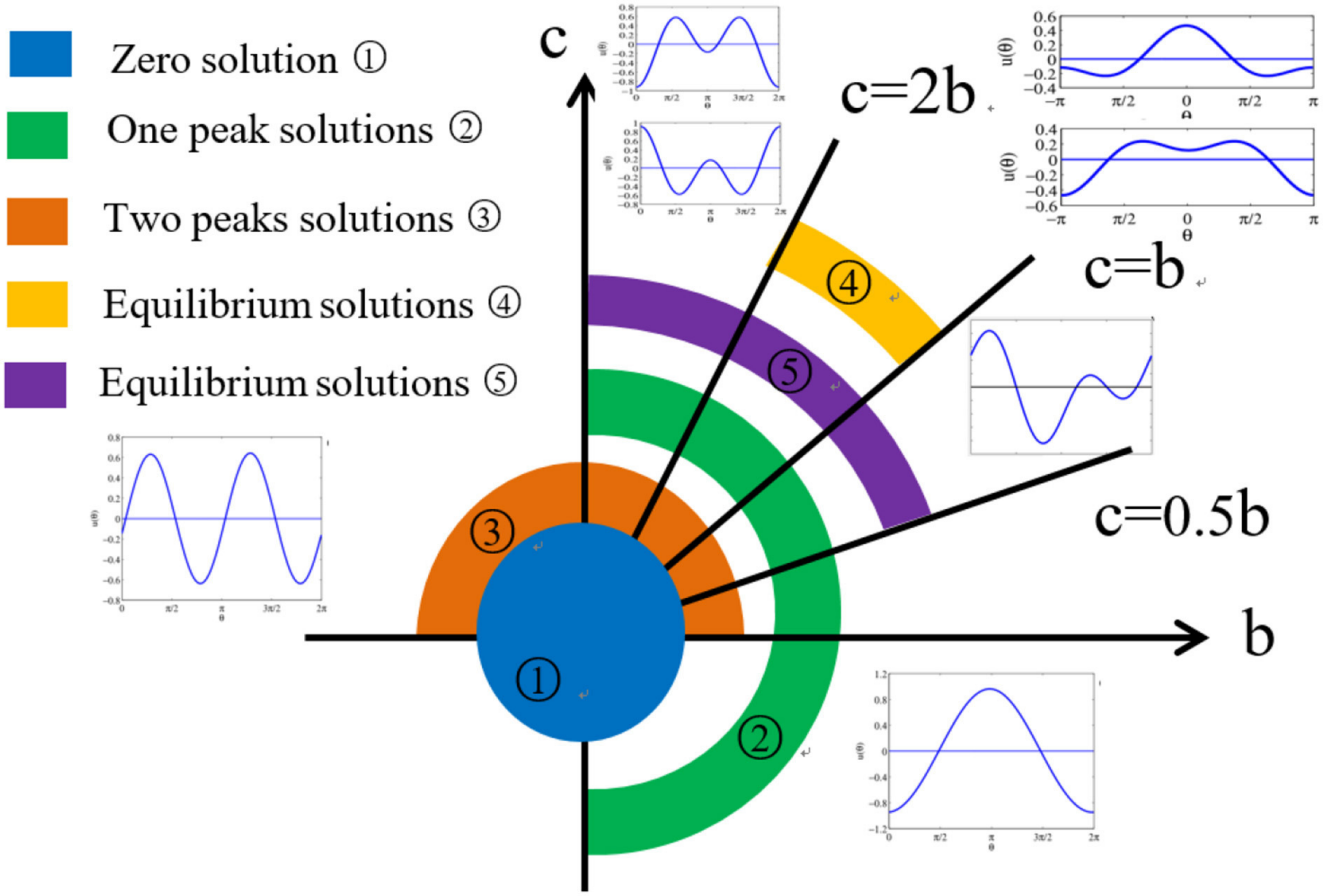
Phase diagram for the distribution of all possible equilibrium solutions in head-direction ring network.

The distribution of all possible equilibrium solutions in system (32) is shown in Figure 9.

#### 3.2.4. Shifting Mechanism

**Figure 10:**
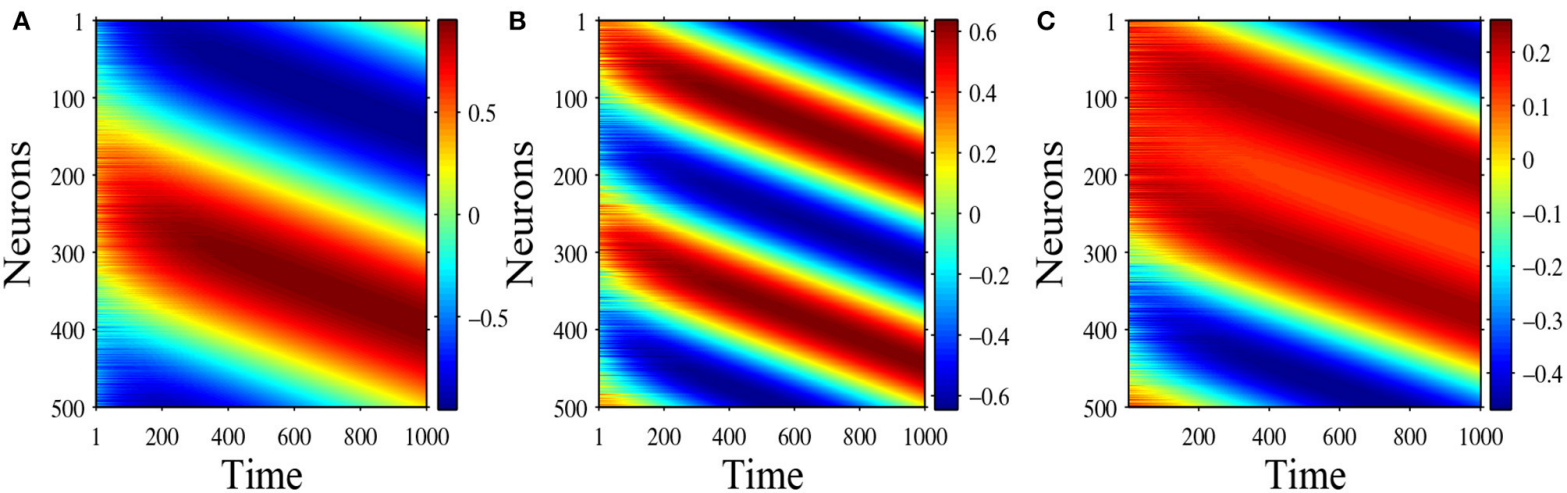
**(A)** Shifting of equilibrium solution $u\left(\theta \right)=\frac{b}{\pi }\text{cos}\left(\theta \right)$, with parameters *a* = 0, *b* = 3, *c* = 2; **(B)** Shifting of equilibrium solution $u\left(\theta \right)=\frac{c}{\pi }\text{cos}\left(2\theta \right)$, with parameters *a* = 0, *b* = 3, *c* = 2; **(C)** Shifting of equilibrium solution $u\left(\theta \right)=\frac{b}{\pi }\sqrt{\frac{c+b}{2c}}\text{cos }\theta -\frac{\sqrt{c^{2}-b^{2}}}{2\pi }\text{cos }2\theta$, with parameters *a* = 0, *b* = 1, *c* = 1.5.

In head-direction ring network, one important biological characteristics is that the attractor bumps can shift in time in response to a head turn. In Figure 10 we select *N* = 500 head-direction cells, and time step Δ*t* = 0.01, and use numerical simulation to demonstrate the shifting mechanism. Following the derivative rule (Zhang, 1996), now the synaptic weight becomes:

(101) $$\begin{array}{l} w_{1}\left(\theta \right)=w\left(\theta \right)+\alpha \frac{dw\left(\theta \right)}{d\theta }, \end{array}$$

where the original synaptic weight is *w*(θ) = *a* + *b* cos θ + *c* cos 2θ, α is the speed of shifting. Here we choose α = 0.2 and initial values are equilibrium solutions plus small disturbances. Figures 10A,B show the shifting mechanism for the one-peaked attractor state $u\left(\theta \right)=\frac{b}{\pi }\text{cos }\theta$ and the two-peaked attractor state $u\left(\theta \right)=\frac{b}{\pi }\text{cos }2\theta$, with parameters *a* = 0, *b* = 3, and *c* = 2. Figure 10C shows the shifting mechanism for the stable special two peaked attractor state $u\left(\theta \right)=\frac{b}{\pi }\sqrt{\frac{c+b}{2c}}\text{cos }\theta -\frac{\sqrt{c^{2}-b^{2}}}{2\pi }\text{cos }2\theta$, with parameters *a* = 0, *b* = 1 and *c* = 1.5.

## 4. Discussion

We have analyzed the equilibrium states of the head-direction ring network and found multiple solutions. Even for the simplest network with step gain function and only two Fourier terms in the weight distribution profile, there are a rich variety of equilibrium solutions. Some of the equilibrium solutions are well known, such as the flat solution and the single-peaked solution, while other solutions are unexpected, such as the asymmetric solutions. In particular, the equilibrium states with two peaks can be generated under many parameter combinations. A necessary condition for the two peaked solution is that the weight profile must have a Fourier component with two peaks. Our analysis reconfirms that it is possible for the ring network to have two stable activity bumps as illustrated in Figure 1. To determine how these different types of equilibrium states depend on the parameters, we have calculated the phase diagram in the parameter space of the ring network with step gain function. Our method can be extended other gain functions such as the standard sigmoidal gain function, as discussed below.

The simple head-direction ring network has some essential dynamical features such as boundedness of state, convergence to stable equilibrium states, and strong dependence of the equilibrium states on the synaptic weight. In this paper our analytical treatment relies on several simplifying assumptions such as truncated Fourier series and step gain function. To generate the equilibrium states, we assume an even weight function *w*(−θ) = *w*(θ) which is equivalent to symmetric reciprocal connection weights because the existence of a Lyapunov function guarantees the stability in this situation. We have only briefly considered the case of asymmetric weights by adding a derivative of the weight profile in order to shift the activity bumps. The parameters in synaptic weight *w*(θ) are the main control parameters that determine the form of an equilibrium state and its stability. As mentioned in section 3, parameter *a* determines the position of the constant solution, parameter *b* determines the existence of one-peaked equilibrium solutions, parameter *c* determines the existence of two-peaked equilibrium solutions, and the values of parameters *a*, *b* and *c* together determine the exact form of an equilibrium state as well as its stability. We find that if the head-direction ring network has one equilibrium solution, then there must exist at least one stable equilibrium solution, i.e., one attractor solution.

**Figure 11:**
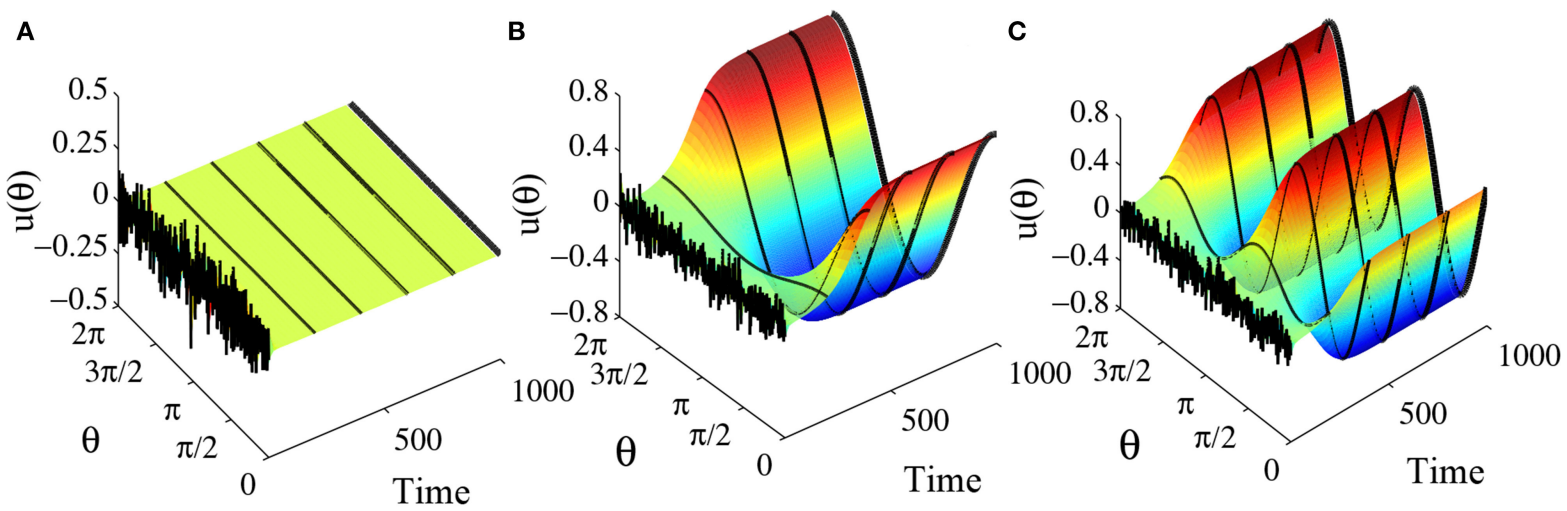
Equilibrium states in the ring network with sigmoidal gain function $g\left(u\right)=\frac{1}{1+e^{-2u}}$. The number of head-direction cells *N* = 50, τ = 1, and time step Δ*t* = 0.001. The synaptic weight function is also *w*(θ) = *b* cos θ + *c* cos 2θ and parameter *a* = 0. **(A)** When *b* = 3.5 and *c* = 3.5, starting from a random initial state, the final state converges to a flat solution *u*(θ) = 0. **(B)** When *b* = 4.5 and *c* = 3.5, starting from a random initial state, the final state converges to a one-peaked equilibrium solution. **(C)** When *b* = 3.5 and *c* = 4.5, starting from a random initial state, the final state converges to a two-peaked equilibrium solution.

**Figure 12:**
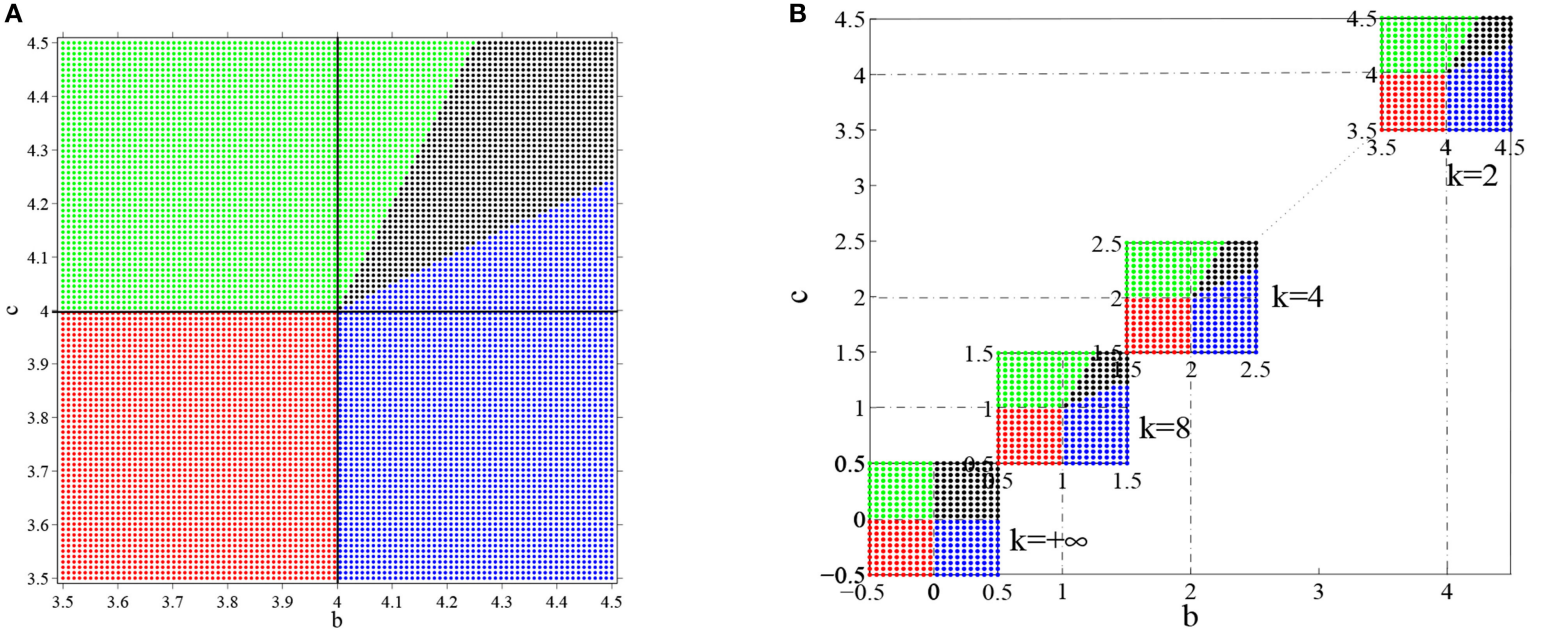
The phase diagram for the distribution of equilibrium states in the ring network with the sigmoidal gain function $g\left(u\right)=\frac{1}{1+e^{-ku}}$. The number of head-direction cells *N* = 50, τ = 1 and time step Δ*t* = 0.001. The synaptic weight function is still *w*(θ) = *b* cos θ + *c* cos 2θ and parameter *a* is 0. The equilibrium state of the system is strongly dependent on parameters *b*, *c* and *k*. **(A)** The parameter space of head-direction ring network when *k* is set to 2 and the initial values are random, *u*_0_ = cosθ and *u*_0_ = cos 2θ. The red domain represents flat equilibrium states, the blue domain represents one-peaked equilibrium states, the green domain represents two-peaked equilibrium states, and black domain allows both one-peaked equilibrium states and two peaked equilibrium states. **(B)** The parameter space with different gain *k*, where *k* = 2, *k* = 4, *k* = 8, and *k* = +∞ and the initial state is same as **(A)**.

The step gain function or Heaviside function may be regarded as the limit of the standard sigmoidal gain function as the slope approaches infinity. When the slope of the sigmoidal gain function is not too small, the behaviors of the ring network are qualitatively quite similar to the network with Heaviside function. For example, consider a ring network with the gain function $g\left(u\right)=\frac{1}{1+e^{-ku}}$ with *k* = 2 and the weight function *w*(θ) = *a* + *b* cos θ + *c* cos 2θ. The parameters *a*, *b* and *c* also determine which kind of equilibrium states the ring network has as well as their stabilities in a manner similar to the network with Heaviside function. The equilibrium state of the system may allow a flat solution, a one-peaked solution and a two-peaked solution, as shown in Figure 11. For *k* = 2, *a* = 0, *b* = 3.5, and *c* = 3.5, the equilibrium state is constant 0, as shown Figure 11A. Actually because parameter *a* = 0, we know that *u*(θ) = 0 is the one and the only constant solution of the system. Figure 11B shows the final state reaching a one-peaked equilibrium state for parameters *a* = 0, *b* = 4.5, and *c* = 3.5. Figure 11C shows a two-peaked equilibrium solution for parameters *a* = 0, *b* = 3.5 and *c* = 4.5. In addition, according to section 3.2.2 we obtain that when

(102) $$\begin{array}{l} \frac{g^{\prime }\left(0\right)b}{2}<1\text{ and }\frac{g^{\prime }\left(0\right)c}{2}<1, \end{array}$$

i.e.,

(103) $$\begin{array}{l} b<\frac{8}{k}\text{ and }c<\frac{8}{k}, \end{array}$$

the constant solution *u*(θ) = 0 is stable. Furthermore $b=\frac{8}{k}$ and $c=\frac{8}{k}$ demarcate the boundary of different parameter domains which contain different types of equilibrium solutions. Figure 12A shows the parameter space for *a* = 0 and *k* = 2. Such phase diagram is obtained by repeated numerical simulation with random initial states. The one-peaked state *u*_0_ = cos θ, the two-peaked state *u*_0_ = cos 2θ, and the flat state *u*(θ) = 0 are all possible equilibrium state, depending on the parameters. In the red domain, *u*(θ) = 0 is the stable equilibrium state. In the blue domain, the state of system converges to the one-peaked equilibrium state. While in the green domain, the state of system converges to the two-peaked equilibrium state, and in black domain the state may converge to either a one-peaked equilibrium state or a two-peaked state, depending on the initial state. In other words, gain *k* determines the boundary of different domains. The phase diagrams for different gain *k* are shown in Figure 12B, where are four cases with *k* = 2, *k* = 4, *k* = 8, and *k* = +∞. We can see that phase diagram changes gradually as the gain slope *k* changes.

Our analysis reveals a diverse set of equilibrium states of the ring network. Although an unstable equilibrium state is not as robust as a stable equilibrium state, it might be useful for generating slow dynamics that slows down around these special states. The existence of stable activity pattern with multiple peaks provides a theoretical foundation for future study of the head-direction system so that new data analysis methods and new experimental designs could be developed to distinguish different computational mechanisms.

## Data Availability Statement

All datasets generated for this study are included in the article/supplementary material.

## Author Contributions

This paper was completed and written by CW under the guidance of KZ.

### Conflict of Interest

The authors declare that the research was conducted in the absence of any commercial or financial relationships that could be construed as a potential conflict of interest.
